# Roles of Akirin1 in early prediction and treatment of graft kidney ischemia‒reperfusion injury

**DOI:** 10.1002/SMMD.20230043

**Published:** 2024-04-02

**Authors:** Xinyuan Li, Guo Chen, Xiang Zhou, Xiang Peng, Mao Li, Daihui Chen, Haitao Yu, Wei Shi, Chunlin Zhang, Yang Li, Zhenwei Feng, Yuhua Mei, Li Li, Simin Liang, Weiyang He, Xin Gou, Jie Li

**Affiliations:** ^1^ Department of Urology The First Affiliated Hospital of Chongqing Medical University Chongqing China; ^2^ CAS Center for Excellence in Molecular Cell Science Shanghai Institute of Biochemistry and Cell Biology Chinese Academy of Sciences Shanghai China; ^3^ Chongqing Key Laboratory of Molecular Oncology and Epigenetics Chongqing China

**Keywords:** composite materials, dielectric materials, doping, electric breakdown, electrical conductivity

## Abstract

Ferroptosis is a predominant contributor to graft kidney ischemia‒reperfusion injury (IRI), resulting in delayed graft function (DGF). However, much less is known about the early predicting biomarkers and therapeutic targets of DGF, especially aiming at ferroptosis. Here, we propose a precise predicting model for DGF, relying on the Akirin1 level in extracellular vesicles (EVs) derived from recipient urine 48 h after kidney transplant. In addition, we decipher a new molecular mechanism whereby Akirin1 induces ferroptosis by strengthening TP53‐mediated suppression of SLC7A11 during the graft kidney IRI process, that is, Akirin1 activates the EGR1/TP53 axis and inhibits MDM2‐mediated TP53 ubiquitination, accordingly upregulating TP53 in two ways. Meanwhile, we present the first evidence that miR‐136‐5p enriched in EVs secreted by human umbilical cord mesenchymal stem cells (UM‐EVs) confers robust protection against ferroptosis and graft kidney IRI by targeted inhibition of Akirin1 but knockout of miR‐136‐5p in UM sharply mitigates the protection of UM‐EVs. The functional and mechanistic regulation of Akirin1 is further corroborated in an allograft kidney transplant model in wild‐type and Akirin1‐knockout mice. In summary, these findings suggest that Akirin1, which prominently induces ferroptosis, is a pivotal biomarker and target for early diagnosis and treatment of graft kidney IRI and DGF after kidney transplant.

## Graphical abstract

Proposed model for how UM‐EVs mitigate ferroptosis and protect against graft kidney IRI. Akirin1, which is prominently elevated in the kidney IRI process, promotes EGR1‐mediated TP53 expression and suppresses MDM2‐mediated TP53 ubiquitination and degradation, accordingly upregulating TP53 in two ways. This further enhances TP53‐mediated suppression of SLC7A11 and thus facilitates ferroptosis. MiR‐136‐5p enriched in UM‐EVs protects against ferroptosis and graft kidney IRI by inhibiting Akirin1.

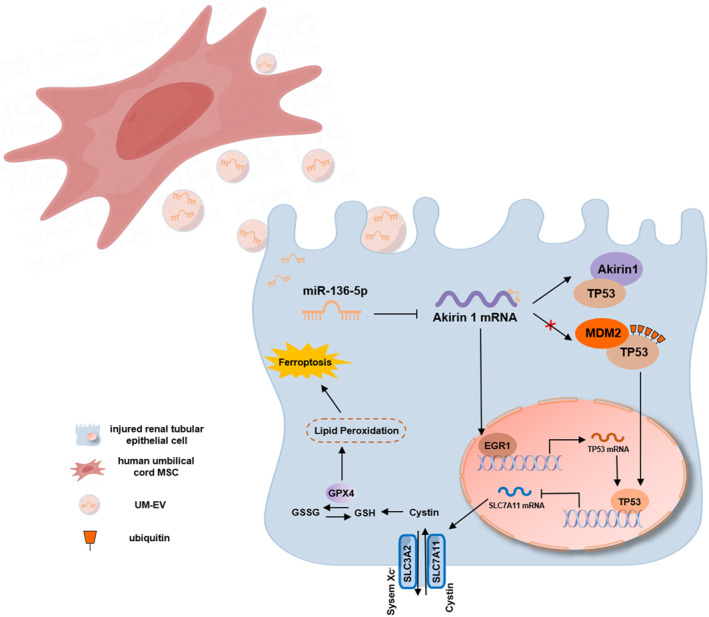

**Key points**

- This study, for the first time, reveals the significance of Akirin1 as a pivotal biomarker and target for the early diagnosis and treatment of graft IRI and DGF after kidney transplant.
- Authors decipher a whole new molecular mechanism by which Akirin1 induces ferroptosis.
- Authors propose a novel therapeutic strategy relying on UM‐EVs for ferroptosis during the renal IRI process.

## 1 INTRODUCTION

Allograft kidney transplant is increasingly becoming the optimal strategy for end‐stage kidney disease; however, the widening gap between supply and demand causes the gradual reliance on marginal donors meeting extended criteria, which affects prognosis to a large extent.^1^, ^2^ Ischemia‒reperfusion injury (IRI), as a predominant contributor to graft tubular necrosis, is an inevitable consequence due to multiple episodes of cold and warm ischemia during donor kidney procurement, storage and transplantation processes in transplant surgery, and thus provokes delayed graft function (DGF).^3^, ^4^ Therefore, improving the prognosis of kidney transplant is leading to a greater focus on a greater mechanistic understanding of graft kidney IRI and identification of precise biomarkers and targets for early prediction and treatment of DGF.

Overwhelming evidence indicates that ferroptosis is the dominant approach driving tubular necrosis during the IRI process.^5^ Ferroptosis is a type of oxidative cell death, which is characterized by the iron accumulation and lipid peroxidation.^6^ Three endogenous antioxidant mechanisms have been deciphered to protect cells from ferroptotic damage, of which the cyst(e)ine/glutathione (GSH)/glutathione peroxidase 4 (GPX4) system involving the heterodimeric cystine glutamate antiporter (system Xc^−^) is well acknowledged as the mainstay restricting ferroptosis by directly regulating cellular cystine uptake, glutamate secretion and GSH biosynthesis and accordingly maintaining cellular redox homeostasis.^7^, ^8^ Genetic and pharmacological inhibition of system Xc^−^ or GPX4 both prominently hypersensitize cells to ferroptosis.^9^ For instance, liproxstatin‐1 was demonstrated to validly suppress lipid oxidation‐induced acute renal failure in GPX4 knockout mice.^10^ Erastin and RSL3‐meidated repression of the cyst(e)ine/GSH/GPX4 axis considerably triggers ferroptosis of kidney vascular smooth muscle cells in chronic kidney disease.^11^ In addition, the soaring number of studies in the last few years revealed a compelling series of molecular mechanisms underlying the antioxidative axis‐modulated ferroptosis. Jiang et al. found that TP53 suppressed cystine uptake and rendered cells more sensitized to ferroptosis by inhibiting the expression of SLC7A11, a key component of the system Xc^‐^.^12^ A study by He et al. validated that long noncoding RNA PVT1 attenuated ferroptosis in hepatocellular carcinoma by competitively interacting with microRNA (miR)‐214‐3p so as to impede the inhibition of GPX4 induced by miR‐214‐3p.^13^ Albeit a growing body of evidence elucidating the enormous predomination of facilitating cyst(e)ine/GSH/GPX4 axis to prevent ferroptosis, exploring more valid regulatory approaches and targets aiming for this antioxidant defense system remain warranted for the prophylaxis and treatment of renal IRI.

Akirin1 is a highly conserved ubiquitously expressed nuclear protein harboring a clear nuclear localization signal and the protein‐protein interaction property.^14^ Acting as a cofactor, Akirin1 has been disclosed that regulate transcriptional activities by establishing a link between multiple transcription factors and chromatin remodeling complexes.^15^ It has been well documented that Akirin1 plays a pivotal role in muscle tissue injury recovery and myogenesis. For example, myostatin decreased Akirin1 at the transcriptional level via ERK/p38MAPK and PI3 kinase pathways, and thus reduced the expression of myogenic regulatory factor 4 and myocyte enhancer factor 2B, inhibiting muscle cell growth and myoblast differentiation.^16^ Intriguingly, a recent study by Rao et al. pinpointed a pro‐oxidative property of Akirin1 which facilitated muscle succinate dehydrogenase expression and activity, a crucial enzyme in the citric acid cycle, and improved the oxidative capacity of skeletal muscle.^17^ These findings raise a novel possibility that Akirin1 participates in the modulation of ferroptosis, which is also under the peroxidation condition.

Mesenchymal stem cells (MSCs), as a heterogeneous subset of stromal cells with self‐renewal and multidirectional differentiation properties, have been well appreciated and exert unequivocal therapeutic effects on IRI via their tissue repair and pro‐regeneration capabilities.^18^ Nevertheless, direct transplantation of MSCs into the injury region, to a large extent, limited the efficacy due to a diverse array of risks such as high heterogeneity, uncertain differentiation and poor survival of grafted MSCs,^19^ at all while, paracrine approaches including extracellular vesicles (EVs) secretion have been corroborated as the mainstay executing mechanisms of MSCs;^20^ therefore, there is increasing interest in the cell‐free therapy relying on MSCs‐derived EVs. EVs derived from human umbilical cord MSCs (UM‐EVs) with the better plasticity, lower immunogenicity and higher proliferation factors showed the huge potential in terms of IRI treatment by regulating diverse cellular processes including ferroptosis.^21^ For instance, UM‐EVs was found that suppressed DMT1 expression by delivering miR‐23a‐3p to inhibit ferroptosis and protect myocardial injury.^22^ In addition, the results from Tan validated that UM‐EVs‐derived BECN1 fostered hepatic stellate cells ferroptosis by suppressing GPX4 expression.^20^ However, few studies have directly disclosed the therapeutic advantages and mechanisms of UM‐EVs for graft IRI after renal transplant, and even fewer reports concerned the protective functions of UM‐EVs from ferroptosis in the renal IRI process.

Here we present the first evidence that Akirin1 was considerably upregulated in the IRI process and induced ferroptosis by potentiating P53‐mediated suppression to the cyst(e)ine/GSH/GPX4 axis, thereby exacerbating tubular necrosis and DGF after renal transplant. In addition, Akirin1 was first demonstrated to increase the transcriptional level of P53, and inhibit MDM2‐mediated ubiquitination by competitively binding to P53. Therapeutically, we identified miR‐136‐5p enriched in UM‐EVs that prominently reduced Akirin1 expression, thus protecting against ferroptosis. These results in the present study, on the one hand, suggest that Akirin1 is a new biomarker and target for early evaluation and treatment of IRI after renal transplant, and on the other hand, propose a novel therapeutic strategy relying on UM‐EVs for ferroptosis during renal IRI process.

## 2 METHODS

### 2.1 Human study

Fifty donor kidneys from thirty‐three donors were transplanted to fifty patients who underwent allograft kidney transplant surgery at the First Affiliated Hospital of Chongqing Medical University (Table 1). The urine samples of these donor‐recipient pairs were obtained for EV separation. Clean and fresh urine of the donor (100 mL) was collected one hour before donor kidney excision, and urine produced from the posttransplant kidney in the recipient (100 mL) was harvested at one and two days after transplant. Written informed consent was obtained from all participants and (or) relatives. We regarded recipients whose SCr levels were more than 400 μmol/L on the 7th day after transplantation as DGF (Supplementary Tables S1 and S2).

**TABLE 1:** Clinical characteristics of kidney pairs included in the study.

Kidney pair ID		Donor age (year)	Doner type	Cold ischemia time	Recipient age (year)	HLA mismatch number	Kidney weight (g)	DDS	Nyberg grade
Doner	Recipient								
1	1	47	ICH	5 h 30 min	54	4	L‐255	20	C
	2				33	2	R‐245	19	B
2	‐	52	ICH	3 h 50 min	46	3	R‐230	24	C
3	1	44	ICH	7 h 15 min	30	5	L‐260	21	C
	2				49	5	R‐260	21	C
4	‐	24	CVM	5 h 15 min	60	2	R‐225	4	A
5	‐	53	ICH	3 h 50 min	44	1	L‐270	24	C
6	1	46	ICH	4 h	33	6	L‐220	20	C
	2				35	2	R‐230	18	B
7	1	34	CHP	8 h 30 min	35	5	L‐255	10	B
	2				55	6	R‐255	10	B
8	‐	55	NCD	4 h	57	3	L‐280	19	B
9	‐	50	ICH	7 h 10 min	54	5	R‐285	24	C
10	‐	29	CVM	5 h 40 min	38	2	R‐240	4	A
11	1	40	ICH	4 h 5 min	34	5	L‐265	18	B
	2				47	2	R‐260	16	B
12	‐	34	CVM	7 h 20 min	54	4	R‐230	10	B
13	1	52	ICH	7 h	48	5	L‐295	27	C
	2				55	4	R‐295	26	C
14	1	36	NCD	6 h 5 min	29	4	L‐270	7	A
	2				33	3	R‐275	7	A
15	‐	61	NCD	9 h 40 min	46	6	L‐275	28	C
16	1	39	ICH	4 h 20 min	51	2	L‐215	9	A
	2				63	4	R‐220	10	B
17	1	35	CVM	5 h 15 min	54	2	L‐240	9	A
	2				67	2	R‐240	9	A
18	1	60	ICH	8 h	39	2	L‐280	27	C
	2				53	4	R‐270	28	C
19	‐	48	ICH	5 h 50 min	56	6	R‐235	18	B
20	1	47	NCD	11 h 5 min	49	1	L‐260	13	B
	2				32	5	L‐260	15	B
21	1	21	CVM	8 h 35 min	28	3	L‐250	5	A
	2				39	6	R‐255	6	A
22	‐	35	NCD	9 h 20 min	59	6	R‐210	8	A
23	1	52	NCD	5 h 10 min	53	3	L‐265	19	B
	2				38	4	R‐265	19	B
24	‐	30	CVM	7 h	41	6	L‐270	11	B
25	1	56	NCD	7 h 30 min	48	5	L‐245	22	C
	2				32	4	R‐240	21	C
26	‐	62	ICH	3 h 40 min	44	2	L‐285	30	D
27	‐	32	CHP	6 h 35 min	47	6	R‐290	8	A
28	‐	41	ICH	6 h 15 min	49	5	R‐240	18	B
29	1	35	CVM	10 h 5 min	54	4	L‐295	10	B
	2				47	1	R‐285	9	A
30	1	49	NCD	9 h	68	5	L‐250	15	B
	2				51	5	R‐250	15	B
31	‐	65	ICH	4 h 25 min	53	6	L‐255	32	D
32	‐	52	NCD	7 h 50 min	40	3	R‐230	19	B
33	1	44	NCD	3 h 15 min	38	5	L‐240	13	B
	2				57	2	R‐240	11	B

Abbreviations: CHP, communicating hydrocephalus; CVM, cerebrovascular malformation; DDS, deceased donor score (Nyberg grade A: 0–9, Nyberg grade B: 10–19, Nyberg grade C: 20–29, Nyberg grade D: 30–39; non‐marginal donor kidney: Nyberg grade A and B, marginal donor kidney: Nyberg grade C and D); HLA, human leucocyte antigen; ICH, intracerebral hemorrhage; L, left kidney; NCD, non‐cerebrovascular disease; R, right kidney.

### 2.2 Mouse model of allograft kidney transplant

Kidney transplant between C57BL/6 donor and recipient mice was conducted as previously described.^23^, ^24^ Donor mice were anesthetized with pentobarbital (50 mg/kg, intraperitoneally) and kept on a thermostatic insulation pad to maintain body temperature. The right donor kidney was excised and stored in 4°C cold Ringer lactate solution for 6 h (cold ischemia). Under general anesthesia, the recipient placed on a heating pad underwent right‐sided nephrectomy, followed by the orthotopic donor‐recipient kidney vascular anastomosis using 10–0 silk sutures within 30 min (warm ischemia). The ureter was then fixed to the bladder lateral wall, and the contralateral native kidney of the recipient was removed. Finally, the abdomen was closed with 4–0 silk suture. According to the guidelines set forth by the American Veterinary Medicine Association, recipient mice were euthanized 2 days after transplant to obtain blood and graft kidney for biochemistry detection and other relative assays.

### 2.3 In vitro IRI model

A type of chemical anoxia/recovery method was used to establish an in vitro IRI model as previously described.^25^, ^26^ HK‐2 cells were first cultivated in glucose‐free medium containing 5 μM antimycin A and 5 mM 2‐deoxyglucose to simulate the ischemic process for 1 h. Then, cells entered a reperfusion phase by replacing the complete medium for 24 or 48 h.

### 2.4 Generation of stable cell lines with lentivirus and CRISPR‒Cas9 systems

To construct cell line with stable overexpression of Akirin1 (AOE), lentivirus vectors (pGLV5/Puro) containing the full‐length cDNA fragments were transfected into HK‐2 cells, followed by puromycin (5 μg/mL) screening for 2 weeks. HK‐2 cells transfected with empty vector were used as a control (WT). In addition, individual guide sequences targeting Akirin1 and microRNA‐136‐5p (miR‐136‐5p) were cloned into pSpCas9 BB‐2A‐Puro (PX459) to establish the knockout cell lines (AKO and miRKO) using the CRISPR‒Cas9 system, as previously described.^27^ The GAL4‐guide sequence was subcloned into the PX459 vector as a control (WT). The sequences of oligonucleotides are listed in Supplementary Table S3.

### 2.5 Other methods and materials

Other methods and materials are provided in the Supporting Information.

## 3 RESULTS

### 3.1 Akirin1 in urine EVs of recipient early predicts DGF after kidney transplant

**FIGURE 1:**
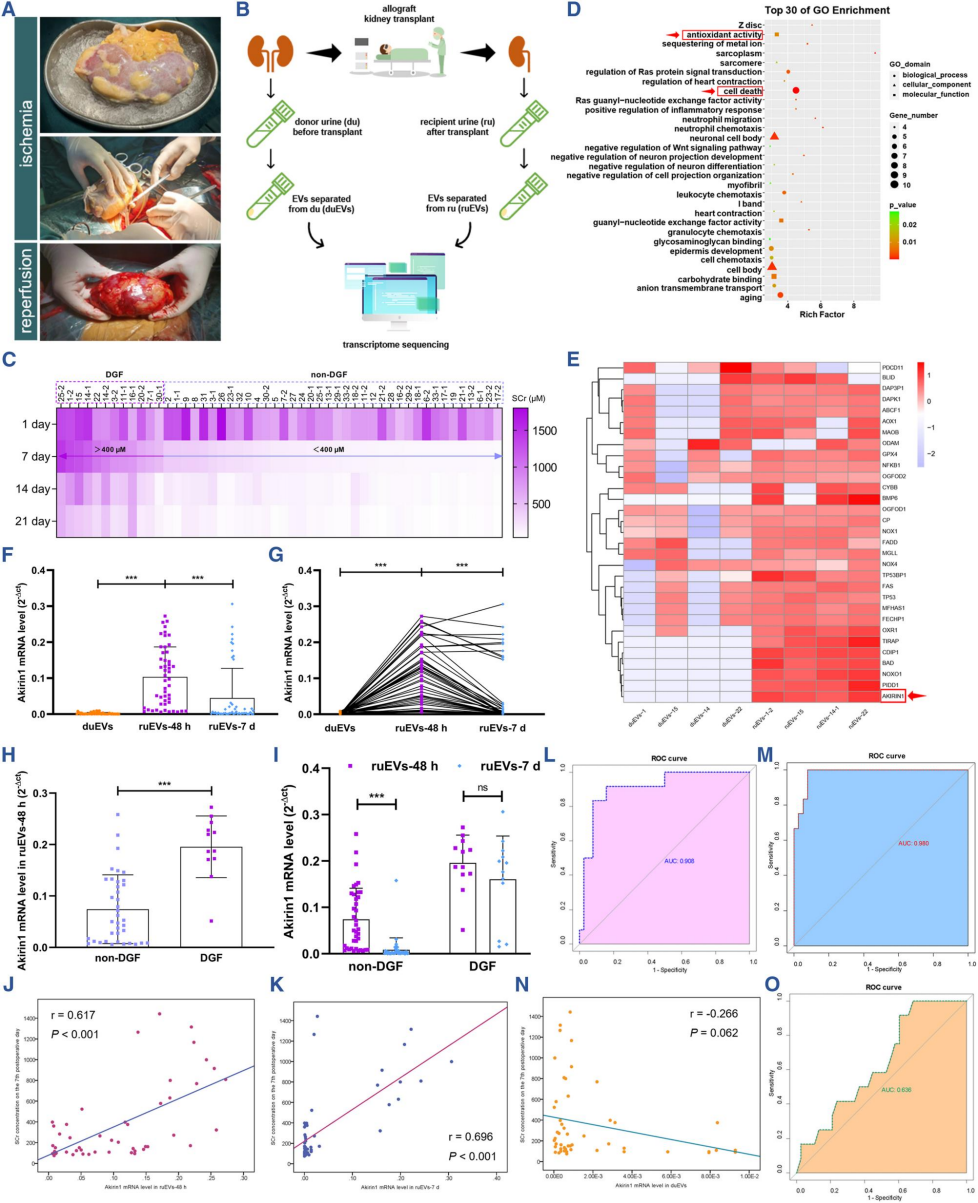
The Akirin1 levels in ruEVs‐48 h confer an unequivocal advantage for early prediction of DGF after kidney transplant (A) Schematic representation of the experimental protocols of allograft kidney transplant. (B) Flowchart delineating the process of uEV extraction and transcriptome sequencing from 50 paired donors and recipients. (C) Heatmap delineating the SCr concentration of DGF and non‐DGF recipients after kidney transplantation (*n* = 50). (D) Gene Ontology analysis of upregulated mRNAs in ruEVs‐48 h (*n* = 4 group^−1^). (E) Cluster heatmap illustrating the differential mRNAs in paired duEVs (*n* = 4) and ruEVs‐48 h (*n* = 4). (F–G) The expression level of Akirin1 was measured by qRT‐PCR in paired duEVs, ruEVs‐48 h and ruEVs‐7 d (*n* = 50), and quantitatively analyzed using one‐way ANOVA followed by Tukey's test and paired two‐tailed Student's *t*‐test respectively. (H–I) qRT‐PCR assays revealing the differential expression of Akirin1 in ruEVs‐48 h and ruEVs‐7 d between non‐DGF (*n* = 38) and DGF recipients (*n* = 12). (J–K) Pearson correlation analysis of SCr concentration on the postoperative 7th day with the Akirin1 level in ruEVs‐48 h (*r* = 0.617, *p* < 0.001, *n* = 50) and Akirin1 level in ruEVs‐7 d (*r* = 0.696, *p* < 0.001, *n* = 50). The ROC curves of the early prediction models for DGF based on the expression level of Akirin1 in ruEVs‐48 h (L) and ruEVs‐7 d (M). (N) Pearson correlation analysis of Akirin1 level in duEVs and SCr concentration on the postoperative 7th day (*r* = −0.266, *p* < 0.062, *n* = 50). (O) ROC curve of the early prediction model for DGF based on the Akirin1 expression level in duEVs. ***p < 0.001, **p < 0.01, and *p < 0.05 represent significant differences between two groups; ns represents no significant difference.

DGF is a common posttransplant adverse event reflecting early acute kidney injury and portending worse graft and patient outcomes. To explore an easy, quick and effective method for early prediction of DGF following kidney transplant and decipher the pathogenesis underlying DGF, we collected 50 pairs of urine EV samples separately derived from 33 donors before kidney transplant (duEVs) and 50 recipients 48 h and 7 d after kidney transplant (ruEVs‐48 h and ruEVs‐7 d), of which 4 pairs of ruEVs‐48 h derived from DGF recipients (SCr≥400 μmoL/L on the postoperative 7th day) and their paired donors were further analyzed by transcriptome sequencing (Figures 1A–C and S1A–C). Interestingly, we found that mRNAs upregulated in the ruEVs‐48 h were mainly enriched for the cell death pathway and closely related to cellular oxidative activity (Figure 1D), of which Akirin1 showed the most striking elevation (Figure 1E). This finding was further corroborated in 50 pairs of duEVs and ruEVs (Figure 1F,G), of which 12 groups of ruEVs were from DGF recipients (Figure 1C). To test if Akirin1 in ruEVs might indicate DGF, we examined Akirin1 levels in ruEVs‐48 h and ruEVs‐7 d of 50 non‐DGF and DGF recipients, and detected a dramatically higher expression of Akirin1 in ruEVs‐48 h of DGF recipients than non‐DGF recipients (Figure 1H). In addition, during the recovery of renal function, the Akirin1 levels in ruEVs of non‐DGF recipients were prominently reduced 7 d after kidney transplant, while we did not detect a significant change in DGF recipients (Figure 1I).

Next, the Pearson correlation analyses suggested significantly positive relevance of SCr concentration on the postoperative 7th day with Akirin1 level in ruEVs‐48 h (*r* = 0.617, *p* < 0.001, *n* = 50, Figure 1J) and ruEVs‐7 d (*r* = 0.696, *p* < 0.001, *n* = 50, Figure 1K). ROC curve analysis further validated that the DGF early predicting model based on the level of Akirin1 mRNA in ruEVs‐48 h (AUC = 0.908, *n* = 50, Figure 1L) and ruEVs‐7 d (AUC = 0.980, *n* = 50, Figure 1M) obtained high sensitivities and specificities. Nevertheless, the Akirin1 level in duEVs showed the poor significance, neither the correlation with SCr concentration (Figure 1N), nor the predicting value for DGF (Figure 1O). Furthermore, we established a validation set containing another 4 DGF and 15 non‐DGF recipients of kidney transplant, and examined Akirin1 levels in ruEVs‐48 h (Supplementary Table S2). ROC curve assay validated the accuracy of Akirin1 for predicting DGF, with the AUC of 0.983 (Figure S1D).

### 3.2 Akirin1 facilitates graft kidney IRI and induces ferroptosis by suppressing SLC7A11

Graft kidney IRI has been well documented to be a major risk factor for DGF. Ferroptosis is the dominant approach driving tubular necrosis during the IRI process. Ferroptosis, a type of oxidative cell death, is characterized by the iron accumulation and lipid peroxidation. Interestingly, the enrichment analyses showed that upregulated mRNAs were closely related to the cell death pathway and cellular oxidative activity (Figure 1D); thus, we investigated the roles of the top five upregulated mRNAs. Silencing expression of Akirin1 led to a prominent decrease in ferroptosis, while siR‐PIDD1, siR‐NOXO1, siR‐BAD and siR‐CDIP1 scarcely affected ferroptosis (Figure S1E–G), implying that Akirin1 probably regulates renal cell ferroptosis during the transplant surgery. In addition, PIDD1, NOXO1, BAD and CDIP1 showed poor significance for predicting DGF (Figure S1H–K). In addition, we detected that the decrease in cell viability caused by Akirin1 overexpression was dramatically reversed when ferroptosis was suppressed, implying that Akirin1 promotes renal IRI by inducing ferroptosis (Figure S1L). However, suppression of ferroptosis by ferrostatin‐1 prominently increased SLC7A11 expression in the renal IRI process but did not affect the expression of caspase 3 and caspase 8 (Figure S1M–O).

**FIGURE 2:**
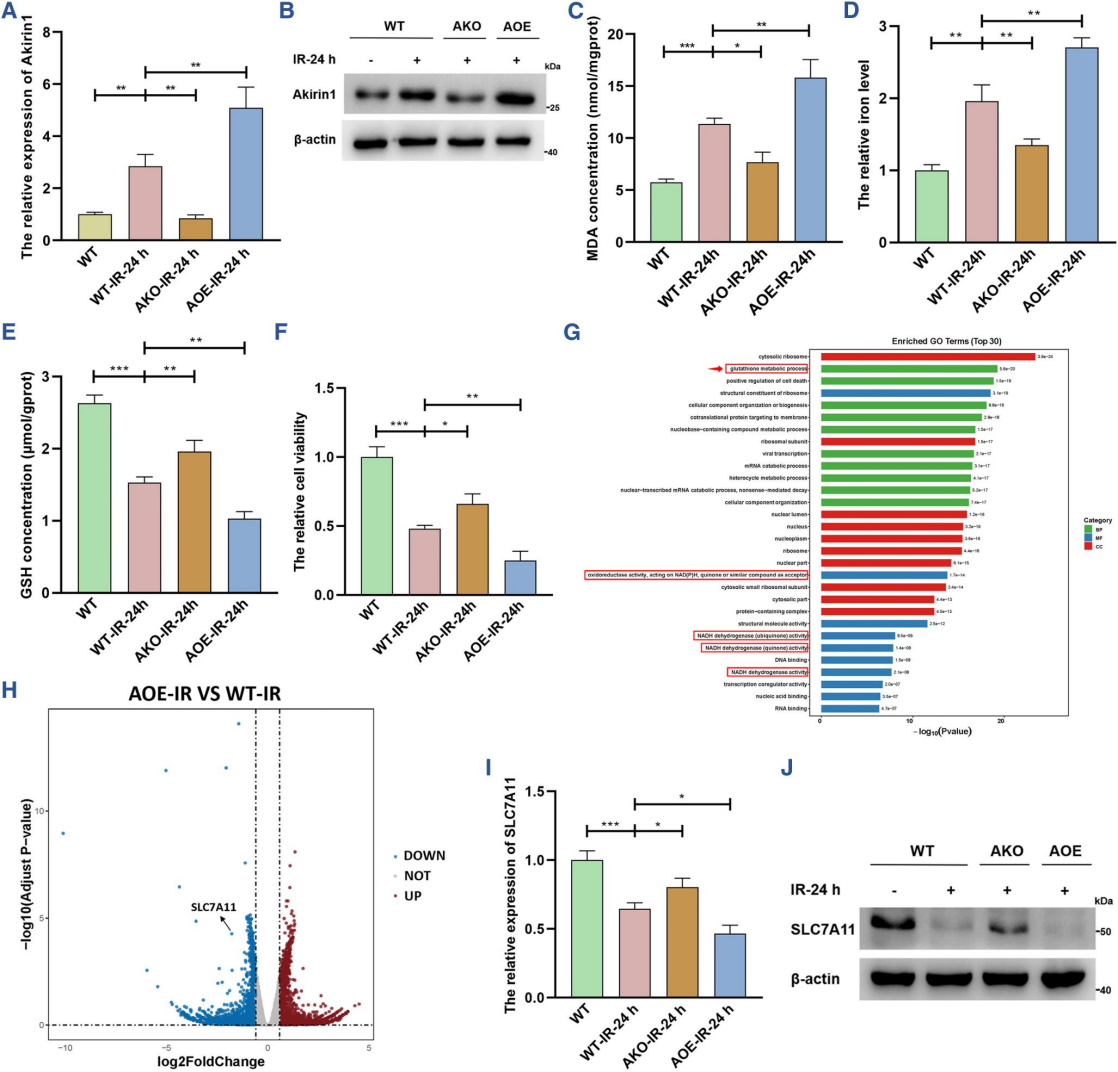
Akirin1 triggers ferroptosis by inhibiting SLC7A11 and thus promotes graft kidney IRI. The transcriptional (A) and translational (B) levels of Akrin1 in different HK‐2 cells were detected by qRT‐PCR and IB assays (*n* = 6 group^−1^), and normalized according to the level of WT; one‐way ANOVA followed by Tukey's test. (C–F) MDA concentration, iron level, GSH concentration and cell viability were detected in HK‐2 cells with different Akirin1 levels (*n* = 6 group^−1^); the results of iron level and cell viability were normalized according to sham; one‐way ANOVA followed by Tukey's test. (G) The top 30 Gene Ontology terms of the differential genes between AOE and WT cells under the IRI condition. (H) Volcano Plot showing the differential genes identified by transcriptome sequencing analysis of WT and AOE cells after IRI, of which SLC7A11 was prominently decreased in AOE group. The transcriptional (I) and translational (J) levels of SLC7A11 in HK‐2 cells with different Akirin1 levels (*n* = 6 group^−1^); one‐way ANOVA followed by Tukey's test. ***p < 0.001, **p < 0.01, and *p < 0.05 represent significant differences between the two groups.

Then, we established in vitro IRI models using HK‐2 cell lines expressing different Akirin1 levels (Figures 2A,B and S2A). First, we detected that the transcriptional and translational levels of Akirin1 were prominently upregulated in the IRI models in a reperfusion time dependent manner (Figure 2B,C). Consistently, ferroptosis was remarkably enhanced following ischemia‐reperfusion, as evidenced by the higher levels of lipid peroxidation and iron and the reductions in GSH and cell viability (Figure S2D–G). Further, when we examined the role of Akirin1 in the IRI process, we found that knockout of Akirin1 sharply suppressed ferroptosis, which was augmented in the IRI model, while ferroptosis was dramatically induced with the ectopic expression of Akirin1 (Figure 2C–F).

To gain further unambiguous insights into the mechanisms by which Akirin1 facilitates ferroptosis, we next performed transcriptome sequencing analysis using WT and AOE cells under IRI conditions and found that Akirin1‐induced differential mRNAs were mainly enriched for the “glutathione metabolic process” which was closely related to cellular antioxidative regulation, and the “positive regulation of cell death” (Figure 2G), which is consistent with the earlier findings in uEVs (Figure 1D). Volcano Plot illustrated that SLC7A11, a pivotal gene promoting glutathione metabolism and thus inhibiting ferroptosis, was strikingly reduced with an increase in Akirin1 (Figure 2H). In contrast, SLC7A11 was considerably elevated at the transcriptional and translational levels in AKO cells (Figure 2I,J). Akirins are also known as relevant mediators of immune function including NF‐kB signaling;^15^ thus, we detected whether Akirin1 regulates the NF‐κB signaling pathway, and the results of western blotting showed that the expression level of NF‐κB was scarcely altered in WT, AOE and AKO cells (Figure S2H). These collective results suggest that Akirin1 prominently induces ferroptosis by suppressing SLC7A11 expression and exacerbates graft kidney IRI.

### 3.3 Akirin1/EGR1/TP53 axis inhibits SLC7A11 and thus promotes ferroptosis in kidney IRI process

**FIGURE 3:**
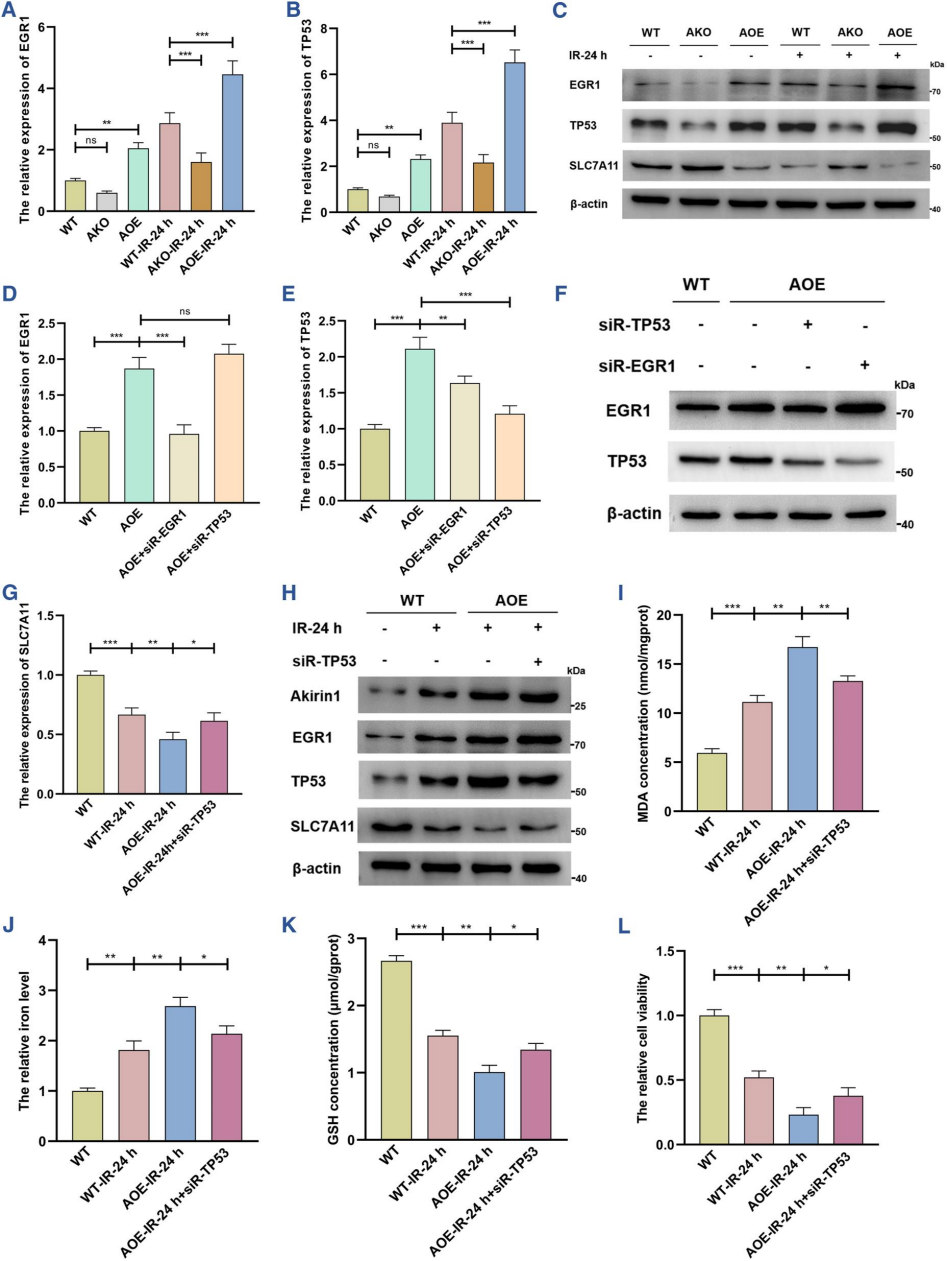
The Akirin1/EGR1/TP53 axis inhibits SLC7A11 and facilitates ferroptosis in the kidney IRI process. The transcriptional levels of EGR1 (A) and TP53 (B) were assessed by qRT‐PCR and normalized by the expression level of WT (*n* = 6 group^−1^); one‐way ANOVA followed by Tukey's test. (C) IB assay examined the translational levels of EGR1, TP53, and SLC7A11 in the IRI model with varying Akirin1 levels (*n* = 6 group^−1^). Different siRNAs were employed to treat HK‐2 cells with different Akirin1 levels, and qRT‐PCR and IB assays detected the transcription (D–E) and translational (F) levels of EGR1 and TP53 (*n* = 6 group^−1^); the results were normalized by the expression level of WT; one‐way ANOVA followed by Tukey's test. The transcriptional and translational levels of SLC7A11 were measured by qRT‐PCR (G) and IB assays (H) in the IRI model with different levels of Akirin1 and TP53 (*n* = 6 group^−1^); the results were normalized by the expression level of WT; one‐way ANOVA followed by Tukey's test. (I–L) MDA concentration, iron level, GSH concentration and cell viability were detected in HK‐2 cells following different treatments (*n* = 6 group^−1^); the result of iron level and cell viability was normalized according to the result of WT; one‐way ANOVA followed by Tukey's test. ***p < 0.001, **p < 0.01, and *p < 0.05 represent significant differences between two groups; ns represents no significant difference.

Next, we focused on the underlying mechanisms by which Akirin1 inhibits SLC7A11. According to the findings of transcriptome sequencing analysis, we further validated that TP53 and EGR1 were increased in a trajectory parallel to that of Akirin1 with the reperfusion time extension (Figure S3A–C). Moreover, the increase of Akirin1 provoked the higher expression of TP53 and EGR1, while SLC7A11 was reduced in the IRI process (Figure 3A–C). Knockout of Akirin1 showed an unequivocally inversed result (Figure 3A–C). To further delineate the regulating roles among TP53, EGR1 and SLC7A11, we separately silenced TP53 and EGR1, and detected that siR‐EGR1 prominently reversed Akirin1‐mediated upregulation of EGR1 and TP53, but siR‐TP53 scarcely affected the transcriptional and translational levels of EGR1 (Figure 3D–F). More importantly, silencing TP53 in AOE cells led to a dramatic increase in SLC7A11 (Figure 3G,H), so as to suppressed Akirin1‐induced ferroptosis in the process of IRI (Figure 3I–L). Together with these findings, we propose a new mechanism by which the Akirin1/EGR1/TP53/SLC7A11 axis promotes ferroptosis.

### 3.4 Akirin1 up‐regulates TP53 by competitively suppressing MDM2‐mediated TP53 ubiquitination

**FIGURE 4:**
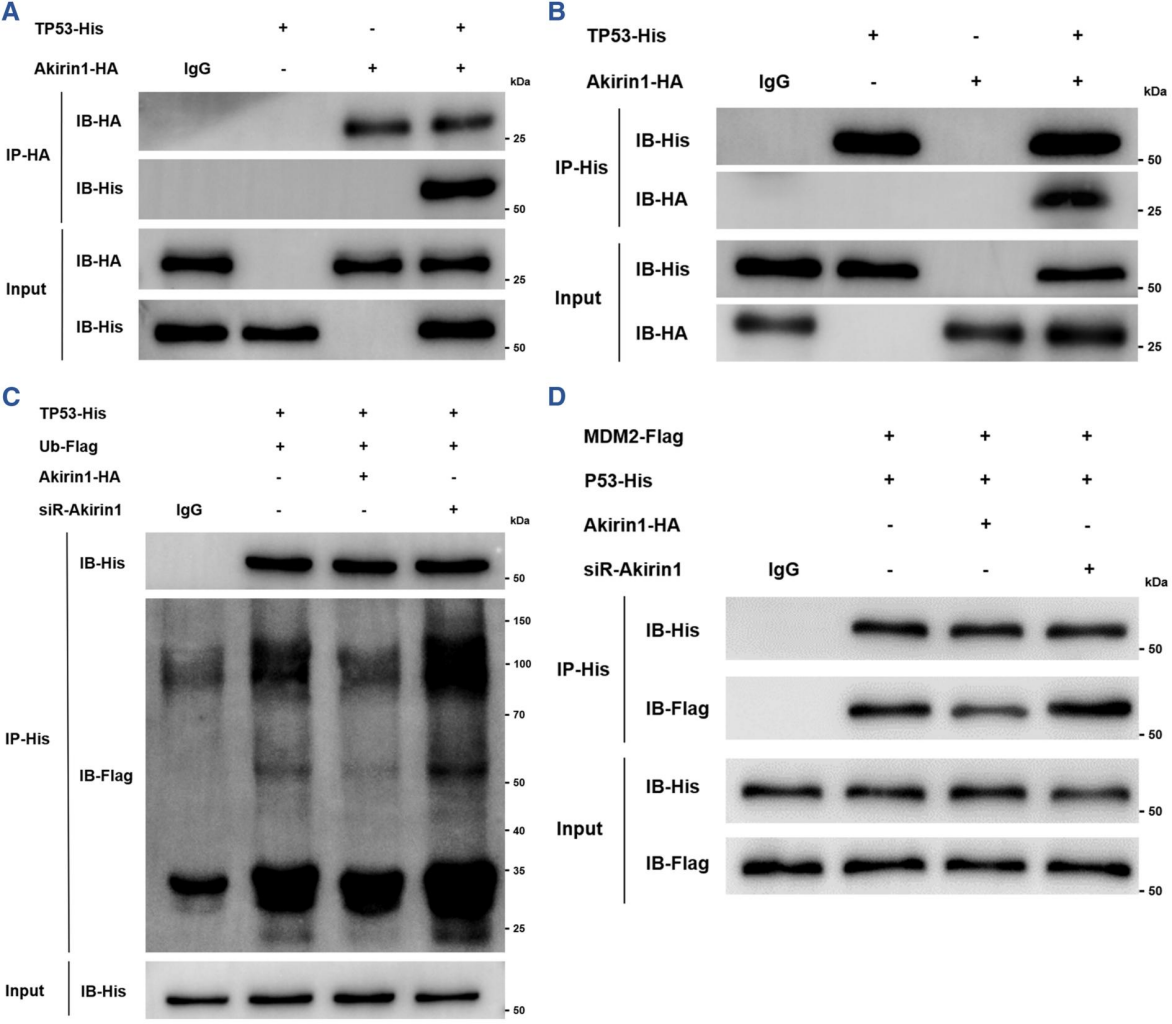
Akirin1 upregulates TP53 by competitively suppressing MDM2‐mediated ubiquitination of TP53. Co‐IP assays of Akirin1‐HA and TP53‐His were examined using an anti‐HA antibody (A) or an anti‐His antibody (B) in 293T cells. (C) The ubiquitination assays of TP53 in 293T cells co‐transfected with Flag‐tagged ubiquitin (Ub‐Flag) with different Akirin1 expression levels. (D) IP assays using an anti‐His antibody in 293T cells co‐transfected with TP53‐His and MDM2‐Flag with different Akirin1 expression levels.

To further investigate whether Akirin1 regulates TP53 through post‐translational mechanisms, we therefore performed co‐IP assays in 293T cells, and the interaction between Akirin1 and TP53 was confirmed at an exogenous level (Figure 4A,B). Ubiquitination has been well documented to be a crucial post‐translational modification for maintenance of p53 stability, both in homeostasis and stress conditions. Interestingly, we found that enhancing Akirin1 expression reduced the interaction between TP53 and Ub, and accordingly antagonized its ubiquitination and proteasome degradation, but siRNA‐Akirin1 reversely provoked a considerable increase in the interaction between TP53 and Ub in 293T cells (Figure 4C).

MDM2 acting as a predominant E3 ubiquitin‐protein ligase mediates ubiquitination of TP53, and the MDM2‐TP53 complex is one of the most crucial hubs bridging other signalling pathways. We therefore posited a probable mechanism whereby Akirin1 inhibits the interaction between TP53 and MDM2. Further co‐IP assays corroborated the hypothesis, as evidenced by a remarkable reduction in MDM2‐TP53 interaction in Akirin1‐overexpressing cells (Figure 4D). Consistently, silence of Akirin1 dramatically facilitated the interaction between TP53 and MDM2 (Figure 4D). In the light of these findings, we reveal a new post‐translational mechanism by which Akirin1 up‐regulates TP53 by competing with MDM2 for TP53 binding and thus reducing MDM2‐mediated TP53 ubiquitination.

### 3.5 The reduction of miR‐136‐5p prominently promotes ferroptosis by targeting Akirin1 in kidney IRI process

**FIGURE 5:**
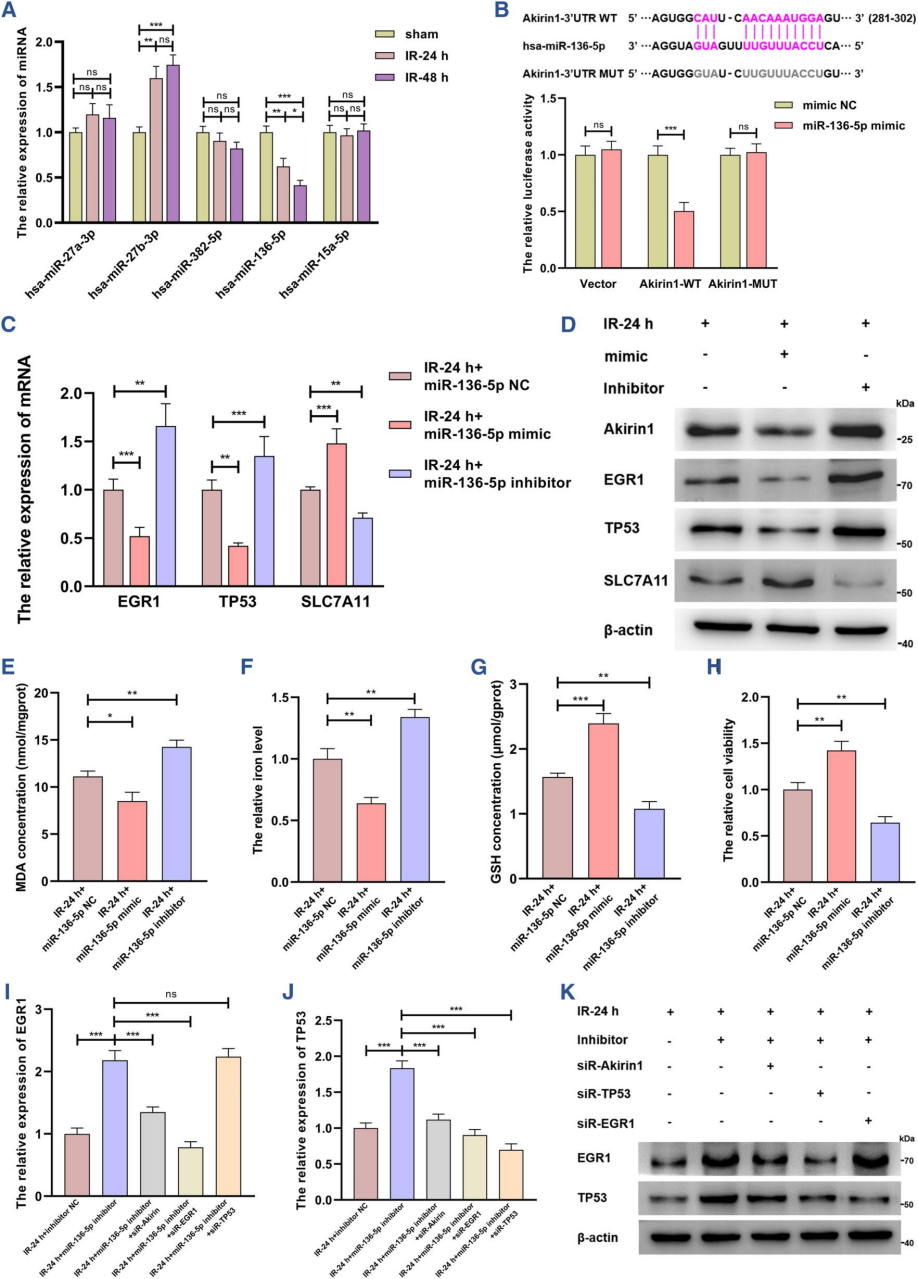
Down‐regulating miR‐136‐5p significantly promotes ferroptosis by inducing Akirin1 in kidney IRI process (A) The relative levels of miRNAs in the 24/48 h IRI model were assessed by qRT‐PCR assays (*n* = 6 group^−1^); the results were normalized according to miRNA level in sham; one‐way ANOVA followed by Tukey's test. (B) Parental (WT) and mutant Akirin1 sequences were cloned into pGL3‐basic plasmid and co‐transfected with miR‐136‐5p into 293 T cells followed by dual luciferase assays (*n* = 3 group^−1^); the results were normalized according to the luciferase activity in 293 T cells transfected with mimic NC; unpaired 2‐tailed Student's *t* test. (C–D) The transcriptional and translational levels of Akirin1, EGR1, TP53, and SLC7A11 were examined by using qRT‐PCR and IB assays in 24 h IRI models with different levels of miR‐136‐5p levels (*n* = 6 group^−1^). (E–H) MDA concentration, iron level, GSH concentration and cell viability were detected in HK‐2 cells following different treatments (*n* = 6 group^−1^); the result of iron level and cell viability was normalized according to the result of WT; one‐way ANOVA followed by Tukey's test. (I–K) The transcriptional and translational levels of Akirin1, EGR1, TP53, and SLC7A11 were evaluated in HK‐2 cells treated with miR‐136‐5p inhibitor and different siRNAs; the results were normalized according to the result of IR‐24+inhibitor NC by one‐way ANOVA followed by Tukey's test. ***p < 0.001, **p < 0.01, and *p < 0.05 represent significant differences between the two groups; ns represents no significant difference.

There is a growing appreciation of the regulatory role of miRNAs in ferroptosis in kidney IRI. We therefore sought to ascertain whether miRNAs regulate Akirin1 and thus undergo cell ferroptosis under IRI conditions. Following the predictive analysis of Akirin1‐related miRNAs in three databases (Figure S4A), we first focused on the top five miRNAs, and subsequently detected that only miR‐27b‐3p and miR‐136‐5p were considerably altered in the kidney IRI process (Figure 5A). After introducing miRNA mimics and inhibitors into HK‐2 cells, Akirin1 was strikingly suppressed by the miR‐136‐5p mimic and increased with the inhibitor treatment, while miR‐27b‐3p showed scarcely any effects on Akirin1 (Figure S4B–E). Besides, Akirin1 did not affect the expression of miR‐136‐5p (Figure S4F). To further corroborate the inhibiting role of miR‐136‐5p on Akirin1, we performed dual luciferase assays with reporters bearing Akirin1 parental cDNA or the mutant at the putative miR‐136‐5p‐binding site, and the results showed that the luciferase activity of Akirin1‐WT, but not MUT, was sharply attenuated by miR‐136‐5p (Figure 5B). In addition, we further analyzed the relevance between the miR‐136‐5p and Akirin 1 levels in ruEVs‐48 h and detected a considerably inverse correlation (*r* = −0.863, *p* < 0.001, *n* = 50, Figure S4G). In addition, the SCr concentration on the postoperative 7th day was also negatively correlated with the miR‐136‐5p level in ruEVs‐48 h (*r* = −0.520, *p* < 0.001, *n* = 50, Figure S4H). These findings strongly imply that miR‐136‐5p was dramatically reduced under kidney IRI conditions, and accordingly facilitated Akirin1 expression.

We next set out to investigate the modulating effects and mechanisms of miR‐136‐5p on ferroptosis. As expected, supplementary with miR‐136‐5p caused an inhibition of the Akirin1/EGR1/TP53 axis and induced SLC7A11 (Figure 5C,D). Accordingly, ferroptosis was prominently ameliorated in the kidney IRI process (Figure 5E–H). To more definitively identify the mechanism of miR‐136‐5p underlying the Akirin1/EGR1/TP53 axis, we separately silenced the encoding genes of Akirin1, EGR1 and TP53. We detected that siRNA‐Akirin1 showed an unequivocally reversing effect on miR‐136‐5p inhibitor‐induced upregulation of EGR1 and TP53 (Figure 5I–K). More persuasively, we found that siR‐EGR1 led to a dramatic reversion of miR‐136‐5p‐mediated TP53 upregulation, but silence of TP53 failed to affect EGR1 expression (Figure 5I–K). These results provide definitive evidence that miR‐136‐5p is strikingly decreased and thus induces ferroptosis by activating the Akirin1/EGR1/TP53/SLC7A11 axis in the kidney IRI process.

### 3.6 UM‐EVs up‐regulate SLC7A11 and inhibit ferroptosis by suppressing Akirin1/EGR1/TP53 axis

**FIGURE 6:**
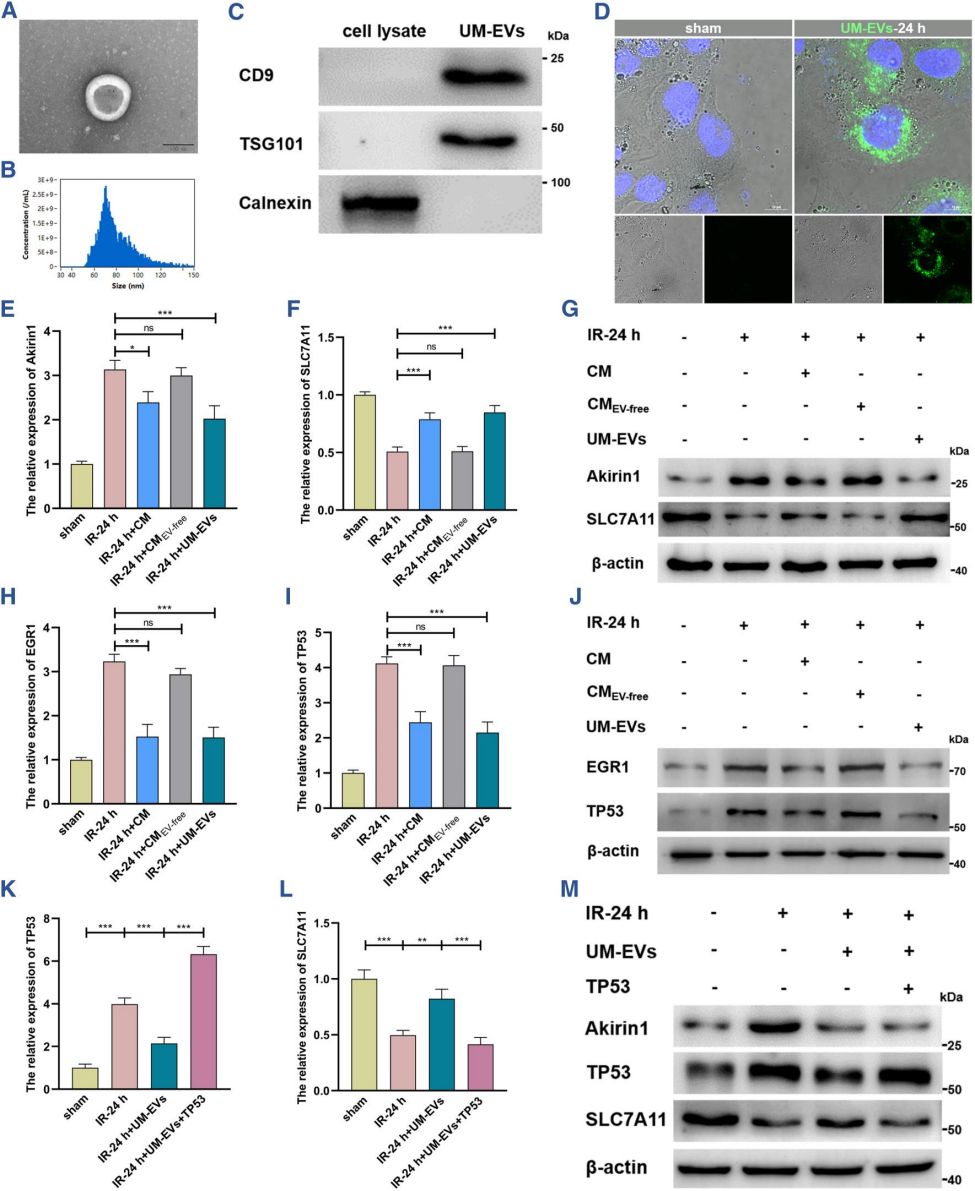
UM‐EVs suppress ferroptosis by inhibiting the Akirin1/EGR1/TP53 axis. (A) UM‐EV morphologies were observed by TEM; scale bar: 100 nm. (B) Flow NanoAnalyzer showing the particle size ranges of UM‐EVs. (C) IB illustrating the expression of three categories of UM‐EV markers (CD9, TSG101, and Calnexin). (D) IF assays recording the intracellular uptake of UM‐EVs (labeled with PKH67, green) by HK‐2 cells; scale bar: 10 μm. (E–J) Different conditioned medium (CM) and UM‐EVs were employed to treat HK‐2 cells, and the transcriptional and translational levels of Akirin1, SLC7A11, EGR1 and TP53 were detected by qRT‐PCR and IB assays (*n* = 6 group^−1^); the results were normalized according to the result of sham; one‐way ANOVA followed by Tukey's test. (K–M) The transcriptional and translational levels of EGR1and TP53 were evaluated in IRI models with varying TP53 levels treated with UM‐EVs or not (*n* = 6 group^−1^); the results were normalized according to the result of sham; one‐way ANOVA followed by Tukey's test. ***p < 0.001, **p < 0.01, and *p < 0.05 represent significant differences between two groups; ns represents no significant difference.

MSCs‐based therapy has received increasing interest in protecting against cell and tissue injury because of the repair and pro‐regeneration functions of MSC‐derived EVs. To investigate the regulatory role of UM‐EVs in Akirin‐1‐induced ferroptosis during kidney IRI process, we used MSC conditioned medium (CM) and UM‐EVs to incubate IRI‐HK‐2 cells (Figure 6A–D), and found that CM caused a prominent reduction in Akirin1 and a significant increase in SLC7A11 in IRI‐HK‐2 cells, while these alterations were sharply inversed when EVs were eliminated from CM (Figure 6E–G). More importantly, we noticed that UM‐EVs played a considerably suppressing role in IRI‐induced Akirin1 upregulation and accordingly facilitated SLC7A11 expression (Figure 6E–G). Consistently, ferroptosis was dramatically inhibited following UM‐EVs treatment in the kidney IRI process, accompanied by the increases in lipid peroxidation and iron levels and downregulation of GSH and cell viability in IRI‐HK‐2 cells (Figure S5A–D).

To gain further mechanistic insights, we further tested whether UM‐EVs promote SLC7A11 by inhibiting the Akirin1/EGR1/TP53 axis. Synergizing with the alteration of Akirin1, we detected striking decreases of EGR1 and TP53 in IRI‐HK‐2 cells following CM and UM‐EVs treatments (Figure 6H–J). Ectopic expression of TP53 sharply reversed UM‐EV‐mediated SLC7A11 upregulation in IRI‐HK‐2 cells but showed scarce effects on Akirin1 (Figure 6K–M). Accordingly, the ferroptosis level was unequivocally elevated (Figure S5E–H). Collectively, the foregoing data suggest that UM‐EVs suppress Akirin1‐induced ferroptosis by regulating the Akirin1/EGR1/TP53/SLC7A11 axis in the kidney IRI process.

### 3.7 UM‐EVs protect against ferroptosis by delivering miR‐136‐5p

**FIGURE 7:**
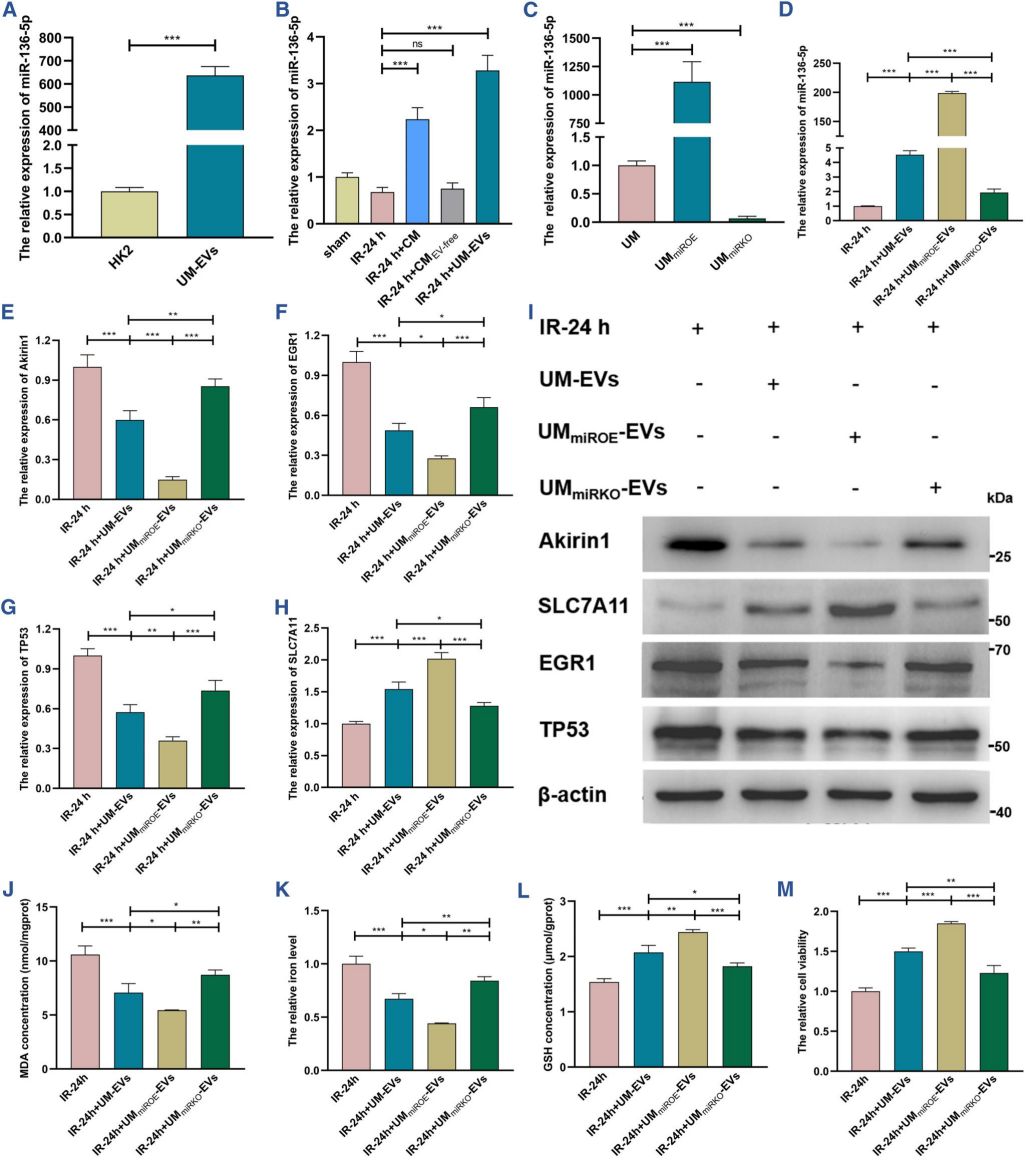
UM‐EVs protect against ferroptosis by delivering miR‐136‐5p (A) The miR‐136‐5p level in UM‐EVs was detected by qRT‐PCR and normalized according to the HK‐2 cells (*n* = 6 group^−1^); unpaired 2‐tailed Student's *t* test. (B) The qRT‐PCR results illustrating the miR‐136‐5p expression in IRI models following different CM and UM‐EVs treatments (*n* = 6 group^−1^); the results were normalized by the level of sham; one‐way ANOVA followed by Tukey's test. (C) The miR‐136‐5p levels in different UMs were assessed by qRT‐PCR and normalized according to the level of UM (*n* = 6 group^−1^); one‐way ANOVA followed by Tukey's test. (D) The miR‐136‐5p levels in IRI models following different UM‐EVs treatments (*n* = 6 group^−1^); the results were normalized according to the level of IR‐24 h; one‐way ANOVA followed by Tukey's test. The transcriptional (E–H) and translational (I) levels of Akirin1, EGR1, TP53 and SLC7A11 in IRI models after different UM‐EVs treatments (*n* = 6 group^−1^); the results were normalized according to the level of IR‐24 h; one‐way ANOVA followed by Tukey's test. (J–M) MDA concentration, iron level, GSH concentration and cell viability were detected in IRI models after different UM‐EVs treatments (*n* = 6 group^−1^); the result of iron level and cell viability was normalized according to the result of IR‐24 h; one‐way ANOVA followed by Tukey's test. ***p < 0.001, **p < 0.01, and *p < 0.05 represent significant differences between two groups; ns represents no significant difference.

Given the promising results of miR‐136‐5p on ameliorating Akirin1‐induced ferroptosis in kidney IRI process and the previously corroborated efficacy of EVs on mediating intercellular communications by delivering signaling molecules including noncoding RNA, we wonder whether UM‐EVs inhibit Akirin1/EGR1/TP53 axis and protect against ferroptosis by delivering miR‐136‐5p. We therefore first examined the prominently higher level of miR‐136‐5p in UM‐EVs compared with HK‐2 cells (Figure 7A), which further provoked a sharp elevation of miR‐136‐5p in IRI‐HK‐2 cells after CM and UM‐EV treatments (Figure 7B). To achieve further mechanistic decipherment, we established the miR‐136‐5p knockout cell line (UM_miRKO_) and over‐expressed cell line (UM_miROE_) (Figure 7C). Compared to UM‐EVs, UM_miRKO_‐derived EVs (UM_miRKO_‐EVs) and UM_miROE_‐EVs notably weakened and increased the miR‐136‐5p level in IRI‐HK‐2 cells, respectively (Figure 7D). Interestingly, knockout of miR‐136‐5p partially reversed the suppression of UM‐EVs on Akirin1/EGR1/TP53 axis, and accordingly upregulated SLC7A11 to some extent, but UM_miROE_‐EVs took the opposite effect on the Akirin1/EGR1/TP53/SLC7A11 axis (Figure 7E–I). Consistently, UM‐EV‐mediated protection against ferroptosis was considerably blunted by the sharp reduction of miR‐136‐5p in UM_miRKO_‐EVs (Figure 7J–M).

### 3.8 UM‐EVs delivering miR‐136‐5p attenuate ferroptosis and graft kidney IRI by modulating Akirin1/EGR1/TP53/SLC7A11 axis during mouse kidney transplantation

**FIGURE 8:**
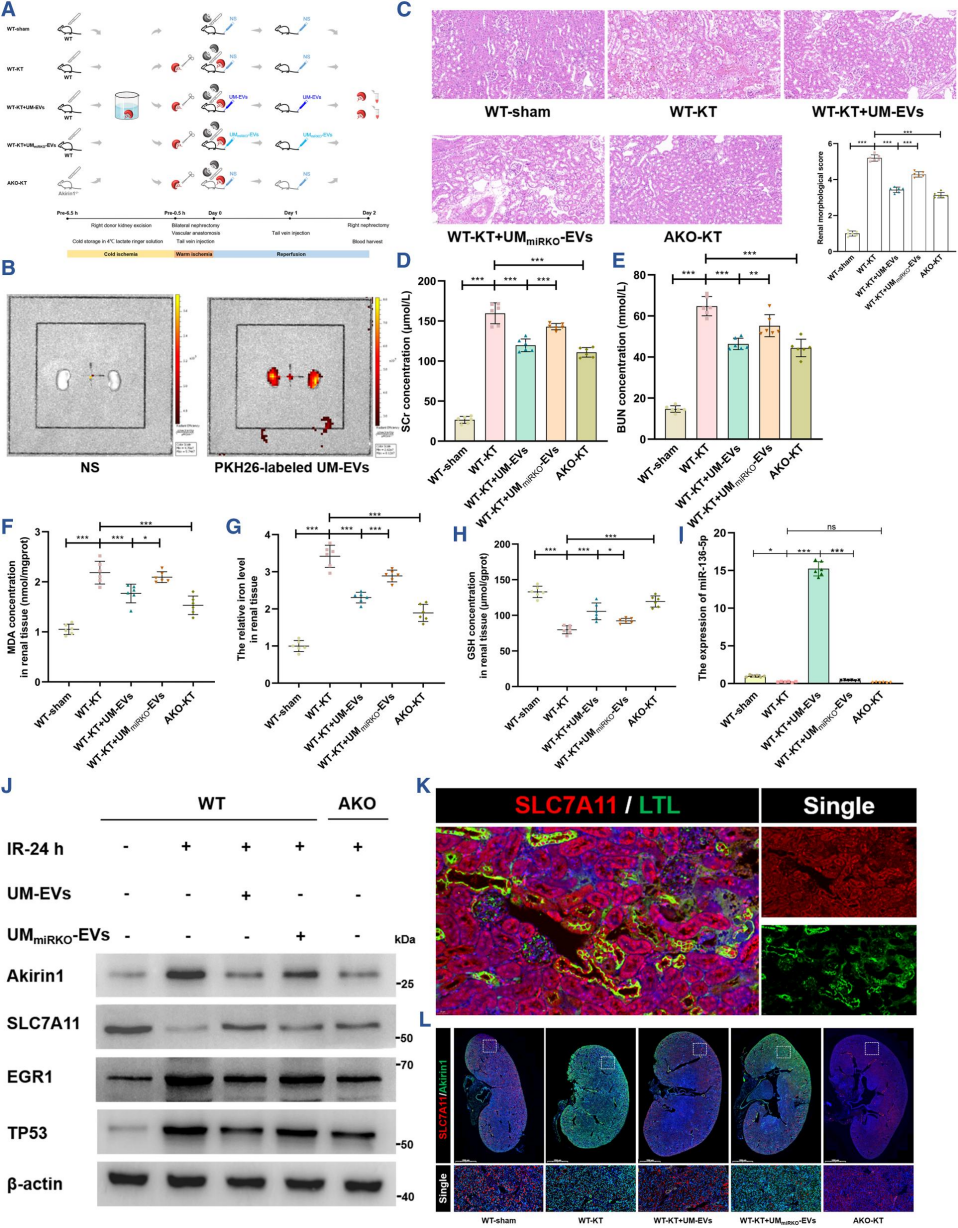
UM‐EVs delivering miR‐136‐5p alleviate ferroptosis and attenuate graft kidney IRI by modulating the Akirin1/EGR1/TP53/SLC7A11 axis during mouse kidney transplantation (A) Flowchart delineating the procedure of allograft kidney transplantation in WT and AKO C57BL/6 mice; for UM‐EVs treatment, 100 μg of UM‐EVs or saline solution (NS) were injected by tail vein 0 and 24 h after kidney transplantation. (B) The tracking of PKH26‐labeled UM‐EVs in mice kidneys, of which the right kidneys were transplanted but the left kidneys were untreated. The right graph shows a pair of kidneys of a recipient mouse which was injected 100 μg PKH26‐labeled UM‐EVs via the tail vein for 6 h, and the left graph shows a pair of kidneys of a recipient mouse which was injected with NS via the tail vein for 6 h. (C) HE staining and renal pathological score of renal tissue slice (*n* = 6 group^−1^); scale bar: 20 μm; one‐way ANOVA followed by Tukey's test. The concentrations of SCr (D) and BUN (E) after various treatments (*n* = 6 group^−1^); one‐way ANOVA followed by Tukey's test. MDA concentration (F), iron level (G) and GSH concentration (H) in graft kidney tissues were normalized according to the level of sham (*n* = 6 group^−1^); one‐way ANOVA followed by Tukey's test. (*n* = 6 group^−1^); (I) The miR‐136‐5p levels in graft kidney tissues were normalized according to the levels of WT‐sham (*n* = 6 group^−1^); one‐way ANOVA followed by Tukey's test. (J) The indicated proteins in graft kidney tissues were assessed by IB assays (*n* = 6 group^−1^). (K) IF assays detecting the expression level and region of LTL (green) and SLC7A11 (red) in WT‐sham renal tissue slices; scale bar: 20 μm for 40X. (L) IF assays detecting the expression level and region of Akirin1 (green) and SLC7A11 (red) in graft renal tissue slices; scale bar: 1000 μm for 1X. ***p < 0.001, **p < 0.01, and *p < 0.05 represent significant differences between two groups.

Inspired by the in vitro potency of UM‐EVs, we next performed the functional and mechanistic validation in wild‐type (WT) and Akirin1^−/−^(AKO) mice models of allograft kidney transplantation (Figure 8A). We first observed that, following tail intravenous injection, the exogenous PKH26‐labeled UM‐EVs were obviously aggregated in a pair of kidneys of a recipient mouse, of which the left kidney was untreated and the right was the transplanted kidney, revealing the similar EV distributions in the native and transplanted kidneys (Figure 8B). We subsequently detected that, consistent with the effects of Akirin1‐knockout, supplementation with exogenous UM‐EVs also attenuated IRI‐induced graft kidney injury in morphology and function, as indicated by H&E staining (Figure 8C) and the SCr and BUN concentrations (Figure 8D–E). However, the protection of UM‐EVs was dramatically weakened by the knockout of miR‐136‐5p in UM_miRKO_‐EVs. In addition, the regulatory effects of UM‐EVs and Akirin1 on ferroptosis in kidney tissues were broadly in line with the morphological and functional modulations (Figure 8F–H).

To yield mechanistic validation, we examined whether the addition of exogenous UM‐EVs rather than UM_miRKO_‐EVs prominently enhanced the miR‐136‐5p level in graft kidney tissues (Figure 8I), accordingly contributing to the downregulation of Akirin1, EGR1 and TP53 and the increase of SLC7A11 (Figure 8J). Similar alteration of the Akirin1/EGR1/TP53/SLC7A11 axis was also detected in AKO mice models (Figure 8J). In addition, the IF assays showed that SLC7A11 was obviously expressed in renal proximal tubules, which were marked by LTL, a marker of renal proximal tubule^28^, ^29^ (Figure 8K), and the exogenous increase of UM‐EVs and endogenous knockout of Akirin1 both considerably up‐regulated SLC7A11 in graft kidneys (Figure 8L). These in vivo findings provide compelling evidence that UM‐EVs inhibit ferroptosis and mitigate graft kidney IRI by regulating the Akirin1/EGR1/TP53/SLC7A11 axis.

## 4 DISCUSSION

Ferroptosis is an unequivocal pathway dominating graft kidney IRI, which further provokes DGF to a large extent.^30^ However, the regulatory mechanisms underlying ferroptosis in the kidney IRI process remain unclear. In addition, there are still lots of open gaps in our current understanding of DGF, including the early predicting methods and therapeutic strategies. In this study, we first disclose the componential variation in uEVs derived from paired donors and recipients of kidney transplants and propose a novel early predicting method for DGF relying on the Akirin1 mRNA level in ruEVs. More importantly, we decipher a whole new molecular mechanism underlying ferroptosis in kidney IRI process, by which Akirin1 up‐regulates TP53 by inducing EGR1 expression and competitively suppressing MDM2‐mediated TP53 ubiquitination and thus causes SLC7A11 reduction. Furthermore, we present the first evidence that UM‐EVs delivering miR‐136‐5p prominently protect against ferroptosis by inhibiting Akirin1, which is sharply up‐regulated in kidney IRI process.

Graft kidney IRI has been well documented to be the leading cause of DGF, further provoking higher rejection rates and worse long‐term outcomes. However, the current understanding is not specific enough for early diagnosis and intervention of DGF, and deciphering deeper mechanisms underlying DGF will help to explore therapeutic targets and identify predicting biomarkers. Hall et al. demonstrated that higher NGAL and IL‐18 levels in recipient urine on the first post‐transplant day were closely correlated with higher DGF rates.^31^ Recent data from Pianta also raised the possibility that urinary increases of IL‐18, KIM‐1 and clusterin can act as clinical features in early predicting DGF.^32^ In the present study, we propose a precise model for the early diagnosis of DGF, relying on the increasing level of Akirin1 in ruEVs on the second post‐transplant day. Moreover, this finding prompted us to further assess in depth whether inhibition of Akirin 1 was a potential therapeutic strategy for graft kidney IRI and DGF.

There has been soaring attention in the last few years for exploring the effective treatments to mitigate graft kidney IRI and minimize DGF. Nevertheless, there are no FDA‐approved therapies to date. Despite hypothermic pump perfusion in graft kidney before transplant has been demonstrated to mitigate IRI to some extent,^33^, ^34^ treatments in recipients, such as superoxide dismutase infusions and anti‐ICAM1 treatment, show an extremely limited effect on IRI protection.^35^, ^36^ Data from two recent clinical studies revealed that using anti‐C5 antibody, silencing RNA to TP53, and inhibiting IL‐6 and HGF expression reduced apoptosis in the graft kidney IRI process.^36^ Nevertheless, increasing evidence suggests that the peroxidative cellular environment during the IRI process prominently facilitates ferroptosis, which plays a predominant role in inducing renal tubular necrosis and leading to DGF. We observed a continuous decrease in the expression of miR‐136‐5p with the prolonged IR time. This could be attributed to the miRNA degradation mechanism mediated by ZSWIM8.^37^ Here, we propose a whole new therapeutic strategy that UM‐EVs delivering miR‐136‐5p protect against ferroptosis and mitigate graft kidney IRI by targeting Akirin1, which is dramatically upregulated in the IRI process.

The Akirin family is widely expressed and plays a variety of roles in embryonic development stages, of which Akirin1 confers an unequivocal advantage for myogenesis.^16^ Mechanistically, Akirin1 has always been demonstrated to activate muscle‐specific RING finger 1 (MuRF‐1), an E3‐ubiquitin ligase, and thus regulate the metabolic activity of skeletal muscle.^17^ In addition, Salerno et al. examined that Akirin1 was dramatically increased at translational and translational levels when the tibialis anterior muscle was injured by notexin, implying that Akirin1 is probably involved in cellular injured regulation.^38^ However, details on the molecular interactions of Akirin‐1 remain incompletely understood, and it is not clear whether it plays a role in the IR‐induced kidney injury. In this study, we first confirmed the predominance of Akirin1 in inducing ferroptosis by strengthening TP53‐mediated suppression of SLC7A11 during the graft kidney IRI process and provided definitive evidence of a considerably facilitating effect of Akirin1 on TP53 expression at both the mRNA and protein levels. Of special importance, we decipher new molecular mechanisms whereby Akirin1 activates the EGR1/TP53 axis and inhibits MDM2‐mediated TP53 ubiquitination, accordingly upregulating TP53 in two ways.

In conclusion, we disclose, for the first time, that Akirin1 is a pivotal predicting biomarker and therapeutic target for DGF after kidney transplant, and decipher a new molecular mechanism by which Akirin1 significantly induces ferroptosis by promoting EGR1‐mediated TP53 expression and suppressing MDM2‐mediated TP53 ubiquitination and degradation. In addition, we corroborated that miR‐136‐5p enriched in UM‐EVs confers robust protection against ferroptosis and graft kidney IRI by targeted inhibition of Akirin1. These findings add to the current repertoire of understanding of ferroptosis in inducing graft kidney IRI and help shape early diagnostic and therapeutic decision‐making for DGF patients after kidney transplant in the context of high DGF incidence rate.

## AUTHOR CONTRIBUTIONS

Conceptualization: Xinyuan Li and Jie Li; Methodology: Xinyuan Li, Guo Chen and Xiang Zhou; Investigation: Xinyuan Li, Guo Chen, Mao Li, Xiang Peng, Wei Shi, Haitao Yu, Chunlin Zhang, Yang Li, Zhenwei Feng, Yuhua Mei and Li Li; Writing‐Original Draft: Xinyuan Li; Funding Acquisition: Jie Li and Xin Gou; Resources: Simin Liang, Daihui Chen, Weiyang He and Xin Gou; Supervision: Jie Li and Xin Gou.

## CONFLICT OF INTEREST STATEMENT

The authors declare that there are no competing interests.

## ETHICS STATEMENT

The study was approved by the Medical Ethics Committee of the First Affiliated Hospital of Chongqing Medical University. We confirmed that none of the organs were procured from executed prisoners and that all donor kidneys were procured after informed consent. All animal experimental protocols conformed to the Chongqing Medical University of Medicine Policy on the Care and Use of Laboratory Animals.

## CONSENT TO PARTICIPATE

The authors confirm that written informed consent for participation was obtained from all patients.

## Supporting information

Supporting Information S1

## Data Availability

The corresponding author can provide the datasets obtained and analyzed during the study upon reasonable request.
