# Supplementary material for: Roles of Akirin1 in early prediction and treatment of graft kidney ischemia‒reperfusion injury
Source: Smart Med. 2024 Apr 2;3(2):e20230043. doi: 10.1002/SMMD.20230043 (PMC11235893; doi:10.1002/SMMD.20230043)
Supplement: Supplementary file 1 — Supporting Information S1 [file SMMD-3-e20230043-s001.pdf]

Supplementary Methods and Materials

Animals

The strain of all mice used in this study is C57BL/6. C57BL/6-Akirin1$^{+/-}$ mice were crossed to generate Akirin1 global knockout mice (C57BL/6-Akirin1$^{-/-}$, AKO). Male AKO mice and wild-type C57BL/6 mice (WT) aged 6 to 8 weeks were used for experiments. All mice were housed at the standardized animal facility in a 12-h light/12-h dark cycle in the Animal Centre of Chongqing Medical University. All animal experimental protocols conformed to the Chongqing Medical University of Medicine Policy on the Care and Use of Laboratory Animals.

Cell culture and treatment

The human proximal tubular cell line (HK-2) and human umbilical cord MSC (UMSC) were purchased from the Cell Bank of the Chinese Academy of Sciences (Shanghai, China) and cultured in Dulbecco's modified Eagle's medium (DMEM)/F12 (Gibco, USA) supplemented with 10% fetal bovine serum (FBS, BioInd, Israel), 100 μg/mL streptomycin (Beyotime, CHN), and 100 U/mL penicillin (Beyotime, CHN) at 37 °C in a humidified atmosphere of 5% CO_2. DMEM (Gibco, USA) was utilized for human embryonic kidney cell line (293T) culture. To establish in vitro sEV-administration models, 30 μg of UM-EVs were added into the complete medium of 10^6 HK-2 cells for 24 h.

Oligonucleotides, plasmids, siRNA and cell transfection

Full-length cDNA, cDNA encoding certain residues and mutant cDNA of tumor protein P53 (TP53), Akirin1, ubiquitin (Ub) and E3 ubiquitin-protein ligase MDM2 were separately subcloned into the pcDNA 3.1 plasmid or pEGFP-N1 plasmid with different tags as indicated. Mimic negative control (NC), miR-27b-3p mimic, miR-136-5p mimic, inhibitor NC, miR-27b-3p inhibitor, miR-136-5p inhibitor, siRNA-Akirin1 (siR-Akirin1), siR-TP53 and siR-EGR1 were synthesized by Tsingke Biotechnology Co., Ltd. (CHN). Plasmids and oligonucleotides were transfected utilizing Lipofectamine 3000 Transfection Reagent (Invitrogen, USA) according to the manufacturer's instructions.

Generation of stable cell lines with lentivirus and CRISPR–Cas9 systems

To construct cell line with stable overexpression of Akirin1 (AOE), lentivirus vectors (pGLV5/Puro) containing the full-length cDNA fragments were transfected into HK-2 cells, followed by puromycin (5 μg/mL) screening for 2 weeks. HK-2 cells transfected with empty vector were used as a control (WT). In addition, individual guide sequences targeting Akirin1 and microRNA-136-5p (miR-136-5p) were cloned into pSpCas9 BB-2A-Puro (PX459) to establish the knockout cell lines (AKO and miRKO) using the CRISPR–Cas9 system. The GAL4-guide sequence was subcloned into the PX459 vector as a control (WT). The sequences of oligonucleotides are listed in Supplementary Table 3.

EVs separation and purification from cell medium and urine

EVs were separated from cell medium by differential ultracentrifugation according to the guidelines of MISEV2018 of the Journal of Extracellular Vesicles. Cells were cultured in EV-free complete medium, of which EVs were depleted by differential ultracentrifugation at 140,000 × g for 18 hours in advance (Type 45 Ti rotor, k-Factor 217.6, Beckman Coulter, USA). For EV separation, cells and cellular debris were first eliminated from the cell medium through sequential centrifugation steps of 300 × g for 10 min, 2,000 × g for 20 min, and 12,000 × g and 4 °C for 30 min, followed by gravitational filtration utilizing a 0.22-μm hydrophilic syringe filter (Millipore, MA, USA). Next, EVs were pelleted from the above resulting medium through two consecutive differential ultracentrifugation separately lasting 70 min at 140,000 × g. Finally, EVs were resuspended in 150 μL of sterile phosphate-buffered saline (PBS) for follow-up experiments or stored at -80 °C. The freezing-thawing cycle was performed not more than one time.

EVs were separated and concentrated from urine by OptiPrep density gradient (ODG) ultracentrifugation. The clean urine samples were briefly precleaned by two successive centrifugation steps at 2,000 × g for 20 min and 12,000 × g and 4 °C for 30 min. Then, the above supernatant was filtered using a 0.22-μm hydrophilic syringe filter. For ODG ultracentrifugation, 50-mL urine samples were first concentrated to 500-800 μL by a 10 kDa centrifugal filter device (Centricon Plus-70, Merck Millipore) and then diluted with Tris buffer (10 mM Tris-HCl, pH: 7.4, 1 mM EDTA and 0.25 M sucrose) to a

volume of 800 μL, which was prepared as a 40% iodixanol suspension by mixing with a 3.2-mL working solution (0.25 M sucrose, 6 mM EDTA, 60 mM Tris-HCl, pH: 7.4). Next, a 17-mL thinwall polypropylene tube (Beckman Coulter, USA) was discontinuously bottom-up filled with 4 mL of 40% iodixanol suspension, 4 mL of 20% iodixanol, 4 mL of 10% iodixanol, 3.5 mL of 5% iodixanol and 1 mL of PBS to build an ODG system. After centrifugation at 100,000 × g and 4 °C for 18 h, the 6th–10th fractions (1 mL) were separately top-down obtained to a new tube, and the final volume was brought to 16 mL with PBS for subsequent centrifugation at 100,000 ×g and 4 °C for 3 h. The urine-derived EV pellets were resuspended in 150 μL of sterile PBS for subsequent analyses or stored at -80 °C.

Transmission electron microscopy

Transmission electron microscopy (TEM, Hitachi-7500, Yokohama, Japan) was used to detect EV morphology. Briefly, EVs were fixed with 0.5% glutaraldehyde solution overnight and dehydrated in absolute ethanol for 10 min. Then, EV sample was dropped onto a 100-mesh formvar-carbon-coated copper grid (TED PELLA, Inc., USA) and stained with 1% phosphotungstic acid.

Flow NanoAnalyzer

Flow NanoAnalyzer in a NanoFCM system (NanoFCM, CHN) was used to measure the high-resolution size distribution of EV.

Tracking of UM-EVs

The UM-EV trace in vitro and in vivo was visualized by using the PKH67 (Sigma–Aldrich, CHN) and PKH26 (Sigma–Aldrich, CHN) label respectively. PKH67-labeled UM-EVs (30 μg) were added to the complete medium of HK-2 cells for 6 h, with PBS supplement serving as a negative control, followed by scanning under a laser confocal microscope (Leica Microsystems AG). In addition, the renal distribution of UM-EVs was imaged 6 h after tail vein injection of PKH26-labeled UM-EVs (100 μg).

Transcriptome sequencing

Paired-end libraries were prepared using a ABclonal mRNA-seq Lib Prep Kit (ABclonal, China) following the manufacturer's instructions. The mRNA was purified

from 1 μg total RNA using oligo (dT) magnetic beads followed by fragmentation carried out using divalent cations at elevated temperatures in ABclonal First Strand Synthesis Reaction Buffer. Then, first-strand cDNAs were synthesized with random hexamer primers and Reverse Transcriptase (RNase H) using mRNA fragments as templates, followed by second-strand cDNA synthesis and adapter ligation. After PCR amplification and purification, the library preparations were sequenced on an Illumina Novaseq 6000 (or MGISEQ-T7) and 150 bp paired-end reads were generated. The data were subsequently used for bioinformatics analysis.

Immunoprecipitation

Preprocessed cells were scratched and lysed in precooled immunoprecipitation (IP) lysis buffer (Beyotime, CHN) containing protease and phosphatase inhibitor cocktails, which were then fully mixed and rotated with 2.5 μg of immunoglobulin G (IgG, CST, USA) or equal amount of target antibody at 4 °C overnight. Next, prewashed protein A/G magnetic beads (MCE, CHN) were used to capture the antigen–antibody complexes at 4 °C for 8 h. Finally, the immunoprecipitated complexes were eluted and quantitatively detected by immunoblotting analysis.

Luciferase reporter assay

293T cells cultured in 96-well culture plates at a density of 2×10^4 cells/well were cotransfected with miR-136-5p pGL3-basic plasmid containing firefly luciferase reporter, an internal control PRL-TK plasmid and pcDNA3.1 plasmid containing cDNA encoding certain residues of Akirin1 or mutant cDNA at a ratio of 5:1:5. The luciferase activity of the transfected cells was measured by the Dual-Luciferase® Reporter Assay

System (Promega, USA), with Renilla luciferase serving as the transfection control.

EV-RNA extraction

After ultracentrifugation, the EV pellet was resuspended in TRIzol reagent (Takara, Japan) according to the manufacturer's instructions. Then, 3M Sodium Acetate solution (pH 5.2) and Dr. GenTLE^{TM} Precipitation Carrier (Takara, Japan) were successively supplemented to EV suspension and fully mixed. Subsequently, double the volume of anhydrous ethanol was added and thoroughly mixed. The mixture was then centrifuged at 12,000 rpm for 15 minutes at 4°C, leaving a white pellet. After air drying, the pellet was dissolved in 20 μL of DEPC water. The EV-RNA sample was stored at -80°C or used directly for subsequent assays.

Quantitative Real-time PCR analysis

Total RNA was extracted from EVs, preprocessed cells or mouse renal tissues according to the instructions in the Takara MiniBest Universal RNA Extraction Kit (Takara, Japan). Purified total RNA was used for reverse transcription with a TransScript® All-in-One First-Strand cDNA Synthesis Supermix (TransGen Biotech, CHN). Quantitative real-time PCR (qRT–PCR) was performed using the SYBR(R) Prime-Script RT–PCR kit (Takara, Japan) and an ABI 7500 Sequence Detection System (Applied Biosystems, USA). Each gene reaction was conducted in triplicate The gene expression level was determined by the internal control β-actin, and was further normalized according to the expression level of the control group.

Immunoblotting

Total protein samples were obtained from cells and mouse renal tissues using

radioimmunoprecipitation lysis buffer (Beyotime, CHN) contiaining protease inhibitor (Thermo Fisher Scientific, USA). Immunoblotting (IB) assays were conducted. Protein was separated by sodium dodecyl sulfate–polyacrylamide gel electrophoresis (SDS–PAGE), followed by blotting on polyvinylidene difluoride (PVDF) membranes (Millipore, USA). The protein blots were visualized with enhanced chemiluminescent substrate (Bio-Rad, USA) and quantified by ImageJ software (NIH, USA).

Immunofluorescence

Renal tissue slices were stained with anti-SLC7A11 and anti-Akrin1 antibodies. Goat anti-rabbit IgG and goat anti-mouse IgG secondary antibodies were utilized for signal visualization. Slides were rinsed and mounted with antifade reagent containing DAPI for nuclear staining. Immunofluorescence (IF) images were photographed using a laser confocal microscope (Leica Microsystems AG).

Renal morphology and function assessment

Hematoxylin and eosin (H&E) staining was used to evaluate the renal histological morphology[30]. Renal tubular injury was assessed by tubular epithelial cell swelling, tubular atrophy and dilatation, loss of brush border, vacuolization and cast formation. Renal function was evaluated by the concentrations of blood urea nitrogen (BUN) and serum creatinine (SCr).

Cell viability assay

Cell viability was detected by using a Cell Counting Kit 8 (CCK-8, Sigma–Aldrich, CHN) according to the manufacturer’s instructions. The absorbance was measured at 450 nm by a microplate reader (Bio-Rad, USA).

Ferroptosis assessment

The lipid peroxidation and iron levels and the glutathione (GSH) concentration were determined to indicate ferroptosis. The malondialdehyde (MDA) concentration was quantified using a lipid peroxidation assay kit (Abcam, UK). The iron level and GSH concentration were detected by using commercial Glutathione Assay Kits (Sigma–Aldrich, CHN).

Statistical analysis

GraphPad Prism 5 (GraphPad software, USA) was utilized for statistical analyses. The ROC curves were drawn and calculated by "pROC" and "rms" packages of R software. Data are presented as the mean ± SD from at least three independent experiments for each group. The statistical significance for comparisons of two groups was determined by unpaired or paired 2-tailed Student's t test. One-way ANOVA followed by Tukey's multiple comparisons test was employed for multigroup comparisons. The linear correlation was evaluated by using Pearson's correlation coefficient. Statistical significance was defined as $P < 0.05$.

Supplementary Figures

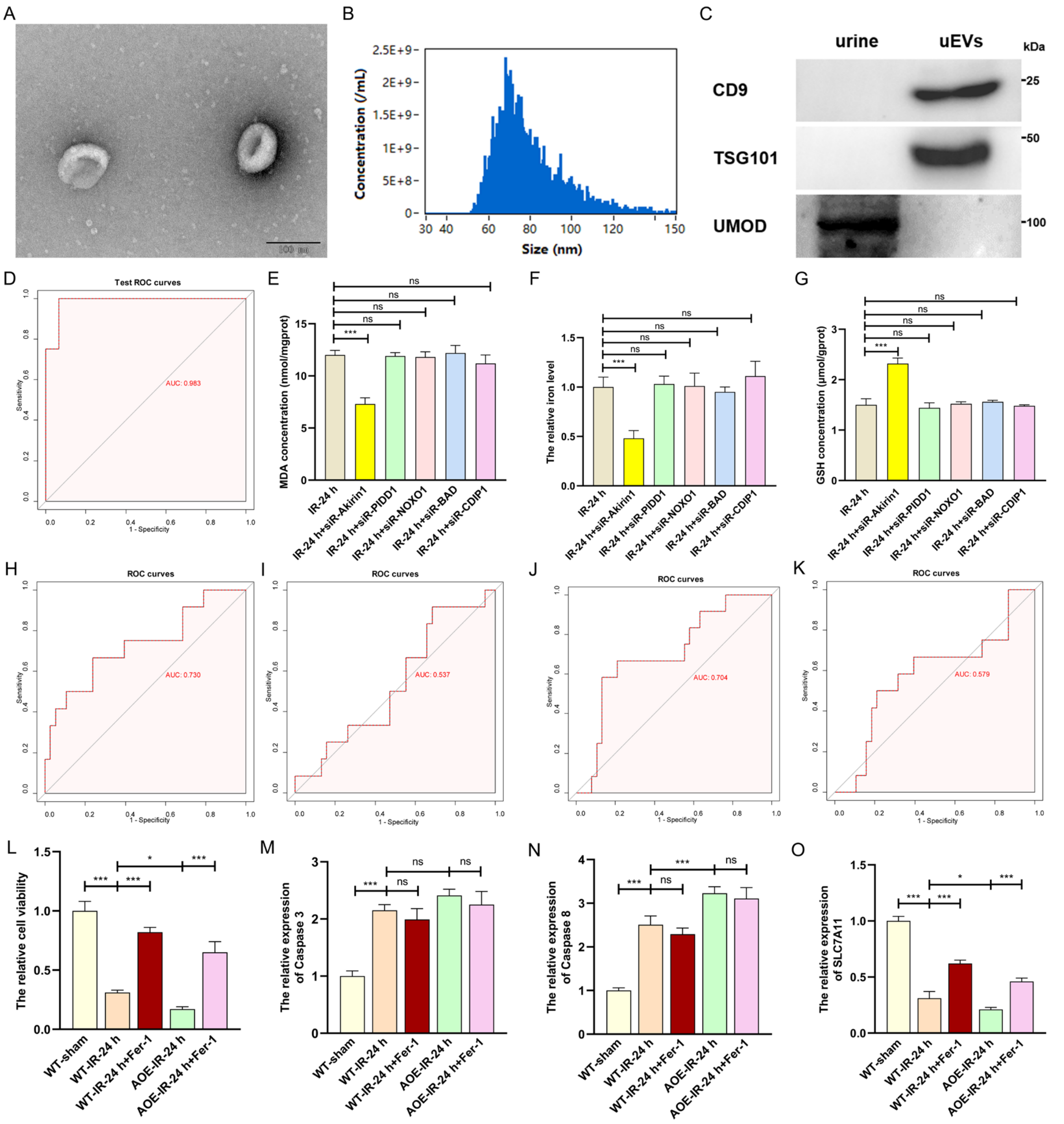

Figure S1. Ferroptosis is prominently induced in kidney IRI process.

(A) The morphologies of uEVs were observed by TEM; scale bar: 100 nm. (B) Flow NanoAnalyzer showing the particle size ranges of uEVs. (C) IB illustrating the expression of three categories of uEV markers (CD9, TSG101, and UMOD). (D) The ROC curve of the early prediction model for DGF based on the expression level of Akirin1 in ruEVs-48 h in a validation set. (E-G) MDA concentration, iron level and GSH concentration were detected in IRI-HK-2 cells following different siRNA treatments (n = 6 group^{-1}); the results were normalized according to IR; one-way ANOVA followed by Tukey’s test. (H-K) The ROC curves of the early prediction

models for DGF based on the expression levels of PIDD1, NOXO1, BAD and CDIP1 in ruEVs-48 h respectively. (L) Cell viability were detected in HK-2 cells with different Akirin1 levels after ferrostatin-1 treatment or not (n = 6 group^{-1}); the results were normalized according to WT; one-way ANOVA followed by Tukey's test. (M-O) The results of qRT-PCR showing the transcriptional level of Caspase 3, Caspase8 and SLC7A11 in HK-2 cells with different Akirin1 levels after ferrostatin-1 treatment or not (n = 6 group^{-1}); the results were normalized according to WT; one-way ANOVA followed by Tukey's test. ***$p < 0.001$, **$p < 0.01$, and *$p < 0.05$ represent significant differences between two groups; ns represents no significant difference.

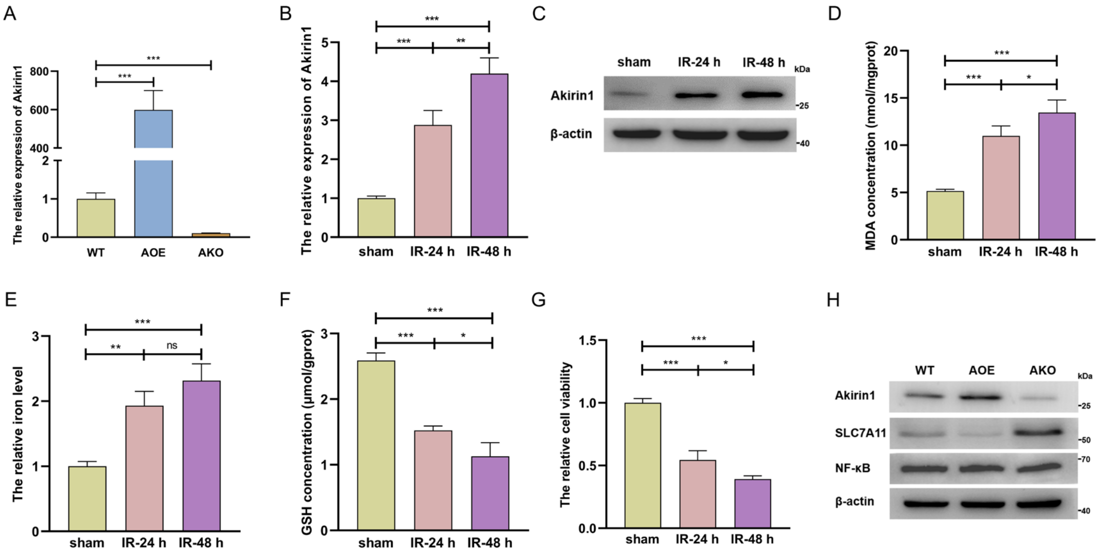

Figure S2. Akirin1 is up-regulated in a time-dependence during kidney IRI process. (A) The Akirin1 levels in HK-2 cell lines (n = 6 group^{-1}); the results were normalized by the result of WT; one-way ANOVA followed by Tukey's test. (B-C) The transcriptional and translational levels of Akirin1 in IRI models with different reperfusion time (n = 6 group^{-1}); the results were normalized by the result of sham; one-way ANOVA followed by Tukey's test. (D-G) MDA concentration, iron level, GSH concentration and cell viability were detected in HK-2 cells with different reperfusion time (n = 6 group^{-1}); the results of iron level and cell viability were normalized according to sham; one-way ANOVA followed by Tukey's test. (H) IB assay examined the translational levels of Akirin1, SLC7A11 and NF-κB in IRI model with varying

Akirin1 levels (n = 6 group^{-1}). ***p < 0.001, **p < 0.01, and *p < 0.05 represent significant differences between two groups.

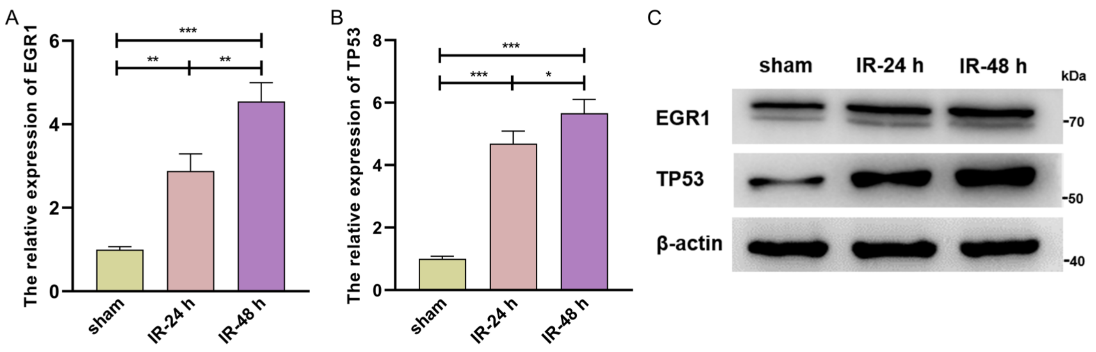

Figure S3. EGR1and TP53 are up-regulated in kidney IRI process.

The transcriptional and translational levels of EGR1 and TP53 were evaluated in IRI models with different reperfusion time (n = 6 group^{-1}); the results were normalized by the result of sham; one-way ANOVA followed by Tukey's test. ***p < 0.001, **p < 0.01, and *p < 0.05 represent significant differences between two groups.

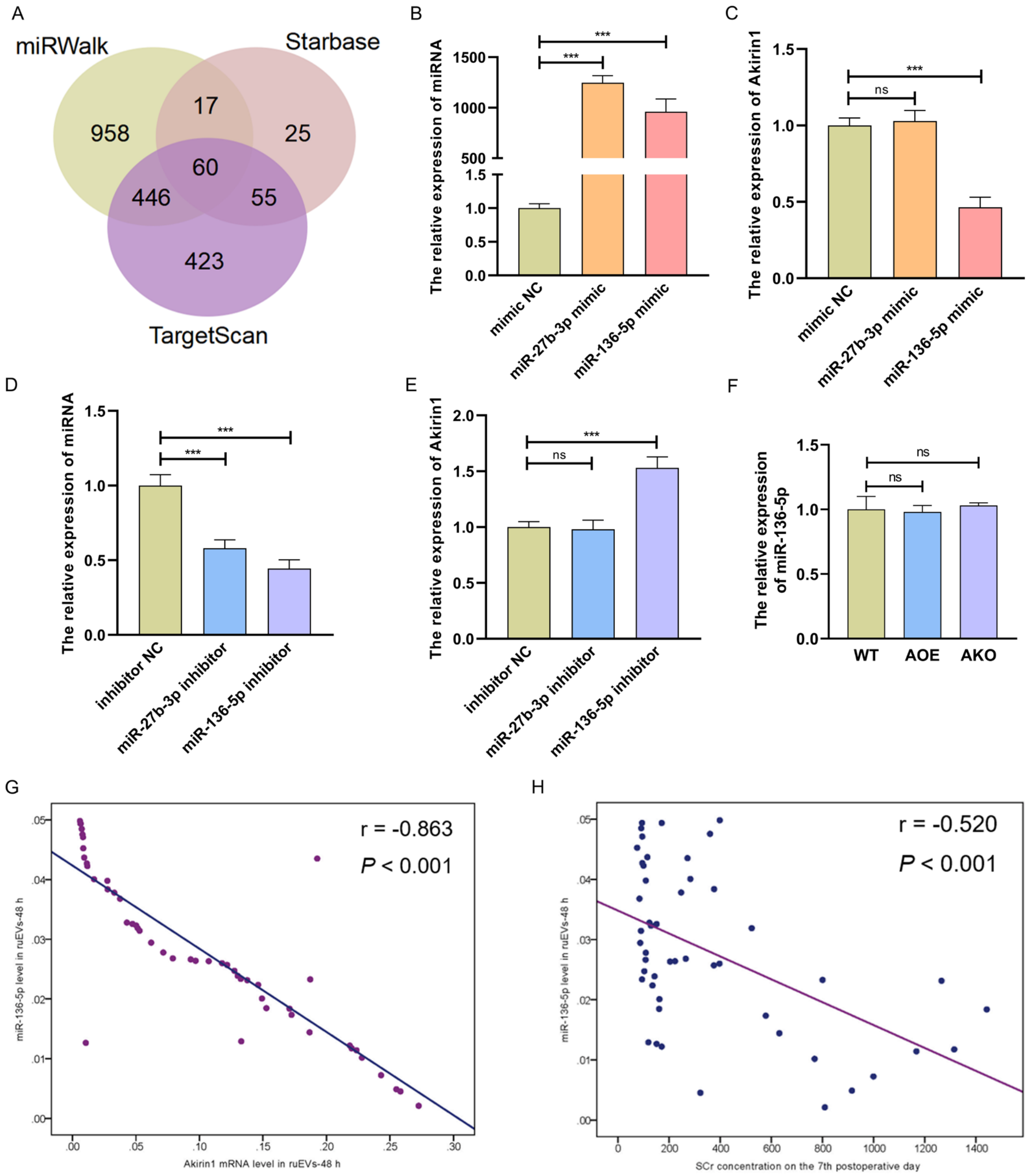

Figure S4. Akirin1 is inhibited by miR-136-5p.

(A) Venn diagram illustrating the intersection of predicted miRNAs binding to Akirin1 in the miRWalk, TargetScan and Starbase database. (B-E) The miRNAs and Akirin1 levels in HK-2 cells treated with mimics and inhibitors of miRNAs (n = 6 $group^{-1}$); the results were normalized according to the result of sham; one-way ANOVA followed by Tukey's test. (F) The miR-136-5p levels in HK-2 cells with different Akirin1 expression; the results were normalized according to WT; one-way ANOVA followed by Tukey's test. (G-H) Pearson correlation analysis of miR-136-5p level in ruEVs-48 h with the Akirin1 level in ruEVs-48 h (r = -0.863, $p < 0.001$, n=50) and SCr concentration on the postoperative 7th day (r = 0.520, $p < 0.001$, n=50). ***$p < 0.001$ represents a significant

difference between two groups; ns represents no significant difference.

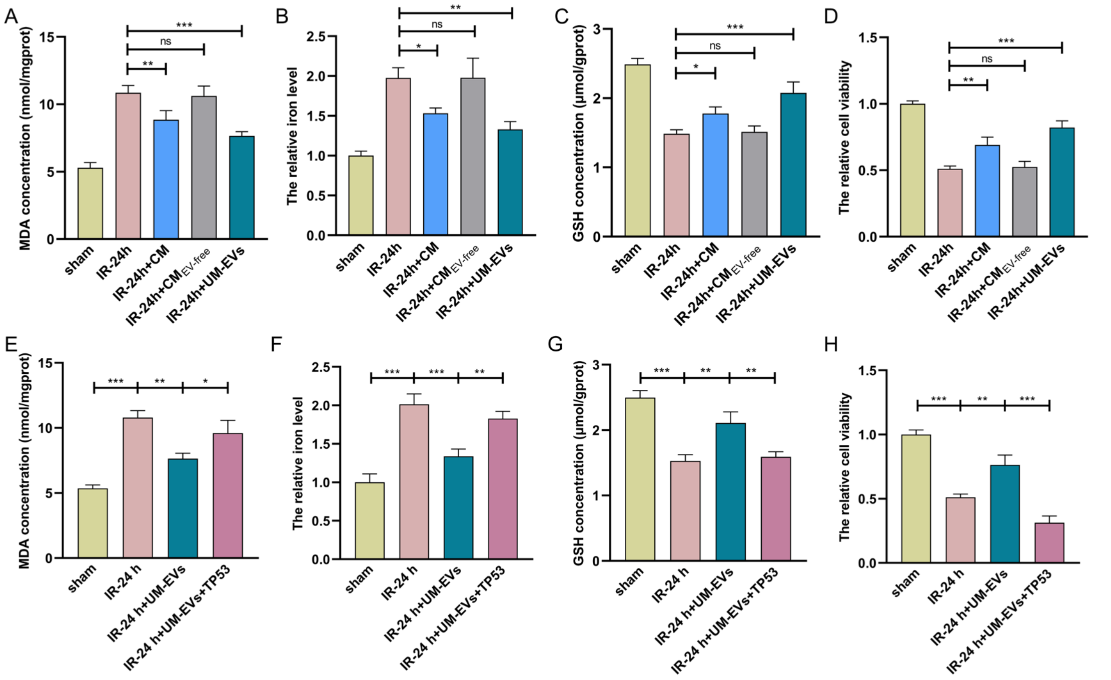

Figure S5. UM-EVs inhibit ferroptosis by suppressing the Akirin1/EGR1/TP53 axis in graft kidney IRI process.

(A-D) MDA concentration, iron level, GSH concentration and cell viability were detected in IRI models following different CM and UM-EVs treatments (n = 6 group^{-1}); the result of iron level and cell viability was normalized according to the result of sham; one-way ANOVA followed by Tukey's test. (E-H) MDA concentration, iron level, GSH concentration and cell viability were evaluated in in IRI models with varying TP53 levels treated with UM-EVs or not (n = 6 group^{-1}); the result of iron level and cell viability was normalized according to the result of sham; one-way ANOVA followed by Tukey's test. ***p < 0.001, **p < 0.01, and *p < 0.05 represent significant differences between two groups; ns represents no significant difference.

Supplementary Table 1: The levels of Akirin1 in ruEVs-48 h and ruEVs-7 d of recipients in the test set

Recipients	Akirin1 in ruEVs-48 h ($2^{-\Delta ct}$)	Akirin1 in ruEVs-7 d ($2^{-\Delta ct}$)	Diagnosis
1-1	0.117829	0.004572	Non-DGF
1-2	0.219865	0.222160	DGF
2	0.005928	0.000348	Non-DGF
3-1	0.258267	0.157790	Non-DGF
3-2	0.272615	0.242615	DGF
4	0.097014	0.002553	Non-DGF
5	0.218395	0.031116	Non-DGF
6-1	0.062019	0.009354	Non-DGF
6-2	0.008315	0.000649	Non-DGF
7-1	0.172513	0.176430	DGF
7-2	0.006537	0.000334	Non-DGF
8	0.121809	0.005510	Non-DGF
9	0.027729	0.001808	Non-DGF
10	0.032986	0.002458	Non-DGF
11-1	0.18726	0.199640	DGF
11-2	0.132906	0.014128	Non-DGF
12	0.009263	0.000184	Non-DGF
13-1	0.130128	0.006250	Non-DGF
13-2	0.052905	0.002347	Non-DGF
14-1	0.223692	0.208380	DGF
14-2	0.255087	0.161660	DGF
15	0.137642	0.020077	DGF
16-1	0.227946	0.152325	DGF
16-2	0.093135	0.003432	Non-DGF
17-1	0.006237	0.000189	Non-DGF
17-2	0.008531	0.000115	Non-DGF
18-1	0.011753	0.000196	Non-DGF
18-2	0.04291	0.001778	Non-DGF
19	0.13255	0.004348	Non-DGF
20-1	0.047326	0.000432	Non-DGF
20-2	0.186719	0.195722	DGF
21-1	0.007326	0.000349	Non-DGF
21-2	0.027536	0.000750	Non-DGF
22	0.243017	0.306320	DGF
23-1	0.192734	0.020628	Non-DGF
23-2	0.037528	0.002229	Non-DGF
24	0.152713	0.002135	Non-DGF
25-1	0.01055	0.002234	Non-DGF
25-2	0.170923	0.026950	DGF
26	0.017032	0.001021	Non-DGF

27	0.149237	0.013467	Non-DGF
28	0.071536	0.006457	Non-DGF
29-1	0.146233	0.014141	Non-DGF
29-2	0.127536	0.002639	Non-DGF
30-1	0.051302	0.015286	DGF
30-2	0.107328	0.000603	Non-DGF
31	0.007913	0.000265	Non-DGF
32	0.079046	0.001997	Non-DGF
33-1	0.011298	0.002500	Non-DGF
33-2	0.050237	0.001056	Non-DGF

Supplementary Table 2: The levels of Akirin1 in ruEVs-48 h of recipients in the validation set

Recipients	Akirin1 in ruEVs-48 h ($2^{-\Delta ct}$)	Diagnosis
1	0.158125	Non-DGF
2	0.387894	DGF
3	0.295122	Non-DGF
4	0.318904	Non-DGF
5	0.208899	Non-DGF
6	0.040763	Non-DGF
7	0.317563	DGF
8	0.185732	Non-DGF
9	0.200215	Non-DGF
10	0.319966	DGF
11	0.163949	Non-DGF
12	0.168410	Non-DGF
13	0.100380	Non-DGF
14	0.339511	DGF
15	0.166490	Non-DGF
16	0.211474	Non-DGF
17	0.124948	Non-DGF
18	0.266814	Non-DGF
19	0.154090	Non-DGF

Supplementary Table 3: The sequence of primer, oligonucleotide, sgRNA and siRNA

Item		Sequence
**β-actin**	Forward	CCTTCCTGGGCATGGAGTC
	Reverse	TGATCTTCATTGTGCTGGGTG
**AKIRIN1**	Forward	GGAAAGTCAACCTCACTCCTCAG
	Reverse	TTGCTCATACTCCTCCCGAATTT
**EGR1**	Forward	TGCTAAAGGGAAAGGGGAA
	Reverse	TTGGGGAAGGGGAAGTG
**TP53**	Forward	TGCGTGTGGAGTATTTGGATG
	Reverse	TGGTACAGTCAGAGCCAACCTC
**SLC7A11**	Forward	ATGCAGTGGCAGTGACCTTT
	Reverse	GGCAACAAAGATCGGAACTG
**has-miR-136-5p**		CGCGACTCCATTTGTTTTGAT
**miR-136-5p mimic**		ACUCCAUUUGUUUUGAUGAUGGA
		CAUCAUCAAAACAAAUGGAGUUU
**miR-136-5p inhibitor**		UCCAUCAUCAAAACAAAUGGAGU
**miR-136-5p-sgRNA-1**		AAAACAAATGGAGTCCTCCGAGG
**miR-136-5p-sgRNA-2**		GTCTCAAATGAGTCTTCAGAGGG
**miR-136-5p-sgRNA-3**		AAACAAATGGAGTCCTCCGAGGG
**Akirin1-sgRNA-1**		GCGGCCCATGGAGTTCGAGGCGG
**Akirin1-sgRNA-2**		TTCAATGCAATGTTGAACTGGGG
**GAL4-sgRNA**		AACGACTAGTTAGGCGTGTA
**siR-Akirin1**		GAAUAUAGUCGUUAUCAGA(dT)(dT)
		UCUGAUAACGACUAUAUUC(dT)(dT)
**siR-EGR1**		GCAGCAGCAGCACCUUCAACC
		UUGAAGGUGCUGCUGCUGCUG
**siR-TP53**		GACUCCAGUGGUAAUCUAC(dT)(dT)
		GUAGAUUACCACUGGAGUC(dT)(dT)

Supplementary Table 4: Antibodies used for Western blotting, IP, and IF.

**Western blotting**			
**Antibodies**	**Source**	**Antibody dilution**	**Identifier**
**Rabbit anti-Akirin1**	ProSci	1:1000	Cat#4799
**Rabbit anti-CD9**	Abcam	1:1000	Cat# ab236630
**Mouse anti-TSG101**	Abcam	1:1000	Cat# ab83
**Rabbit anti-HA**	Cell Signaling Technology	1:1000	Cat#3724
**Rabbit anti- DYKDDDDK**	Cell Signaling Technology	1:1000	Cat#14793
**Rabbit anti- His**	Cell Signaling Technology	1:1000	Cat#12698
**Rabbit anti-Calnexin**	Cell Signaling Technology	1:1000	Cat# 2679
**Rabbit SLC7A11/Xct**	Proteintech	1:1000	26864-1-AP
**Mouse anti-GPX4**	Proteintech	1:2000	67763-1-Ig
**Rabbit anti-EGR1**	Proteintech	1:2000	22008-1-AP
**Rabbit anti-TP53**	Proteintech	1:5000	10442-1-AP
**Mouse anti-β-actin**	Proteintech	1:5000	Cat# 66009-1-Ig
**Rabbit anti-UMOD**	R&D Systems	1:1000	Cat# MAB5175
**IP**			
**Mouse anti-His-Tag**	Abmart	1:100	Cat# M20001
**Mouse anti-HA-Tag**	Abmart	1:100	Cat# M20003
**Protein A/G magnetic beads**	MCE		Cat# HY-K0202
**IF**			
**SLC7A11/Xct**	Proteintech	1:500	26864-1-AP
**Anti-AKIRIN1**	Sigma	1:500	HPA051871-25UL
**DAPI**	Cell Signaling Technology	1:100	Cat# 4083
**Goat anti-Rabbit IgG (Alexa Fluor® 488 Conjugate)**	Cell Signaling Technology	1:500	Cat# 4412

Supplementary Table 5: Chemicals, Critical Commercial Assays and cell lines.

Item	Source	Identifier
**2× Taq PCR Green Mix**	Takara	RR820A
**RNAiso Plus**	Takara	9108
**MG132**	Sigma–Aldrich	M7449
**CHX**	Sigma–Aldrich	5087390001
**D-2-Deoxyglucose**	Sigma–Aldrich	D8375
**Antimycin A**	Sigma–Aldrich	A8674
**Lipofectamine 2000**	Invitrogen	11668019
**Lipofectamine 3000**	Invitrogen	L3000015
**Critical Commercial Assays**		
**BCA protein assay Kit**	Thermo Fisher Scientific	23227
**DAB kit**	Thermo Fisher Scientific	34002
**Cell lysis buffer for Western and IP**	Beyotime	P0013
**RIPA**	Beyotime	P0013B
**PMSF**	Beyotime	ST506
**4% Paraformaldehyde**	Beyotime	P0099
**Penicillin-Streptomycin**	Beyotime	C0223
**Lipid Peroxidation MDA Assay Kit**	Beyotime	S0131S
**Cell Counting Kit-8**	Dojindo	CK04
**Dual-Luciferase® Reporter Assay System**	Promega	E1910
**PVDF membranes**	Millipore	ISEQ00010
**PrimeScript® RT reagent Kit**	Takara	RR047A
**Mir-X miRNA First-Strand Synthesis Kit**	Takara	638313
**Protease Inhibitor Cocktail**	Bimake	B14001
**Dulbecco's modified Eagle's medium**	Gibco	C11995500BT
**DMEM/F12**	Gibco	11320033
**Foetal bovine serum**	BioInd	04-001-1A
**Bovine serum albumin**	Sigma–Aldrich	9048-46-8
**PKH67**	Sigma–Aldrich	MINI67
**PKH26**	Sigma–Aldrich	MINI26
**EDTA**	Solarbio	E1170
**Transwell chamber**	Corning	3450
**Reduced Glutathione (GSH) Colorimetric Assay Kit**	Elabscience	E-BC-K030-M
**Ferrous Iron Colorimetric Assay Kit**	Elabscience	E-BC-K773-M
**HK-2 cell line**	Cell Bank of the Chinese Academy of Sciences	SCSP-511
**293T cell line**	Cell Bank of the Chinese Academy of Sciences	GNHu17
